# Epigenetic Regulations in Neuropsychiatric Disorders

**DOI:** 10.3389/fgene.2019.00268

**Published:** 2019-04-04

**Authors:** Janise N. Kuehner, Emily C. Bruggeman, Zhexing Wen, Bing Yao

**Affiliations:** ^1^Department of Human Genetics, Emory University School of Medicine, Atlanta, GA, United States; ^2^Department of Psychiatry and Behavioral Sciences, Emory University School of Medicine, Atlanta, GA, United States; ^3^Department of Cell Biology, Emory University School of Medicine, Atlanta, GA, United States; ^4^Department of Neurology, Emory University School of Medicine, Atlanta, GA, United States

**Keywords:** epigenetics, neuropsychiatric disorders, DNA methylation, Rett syndrome, Fragile X syndrome, autism spectrum disorders, schizophrenia, major depressive disorders

## Abstract

Precise genetic and epigenetic spatiotemporal regulation of gene expression is critical for proper brain development, function and circuitry formation in the mammalian central nervous system. Neuronal differentiation processes are tightly regulated by epigenetic mechanisms including DNA methylation, histone modifications, chromatin remodelers and non-coding RNAs. Dysregulation of any of these pathways is detrimental to normal neuronal development and functions, which can result in devastating neuropsychiatric disorders, such as depression, schizophrenia and autism spectrum disorders. In this review, we focus on the current understanding of epigenetic regulations in brain development and functions, as well as their implications in neuropsychiatric disorders.

## Introduction

**FIGURE 1:**
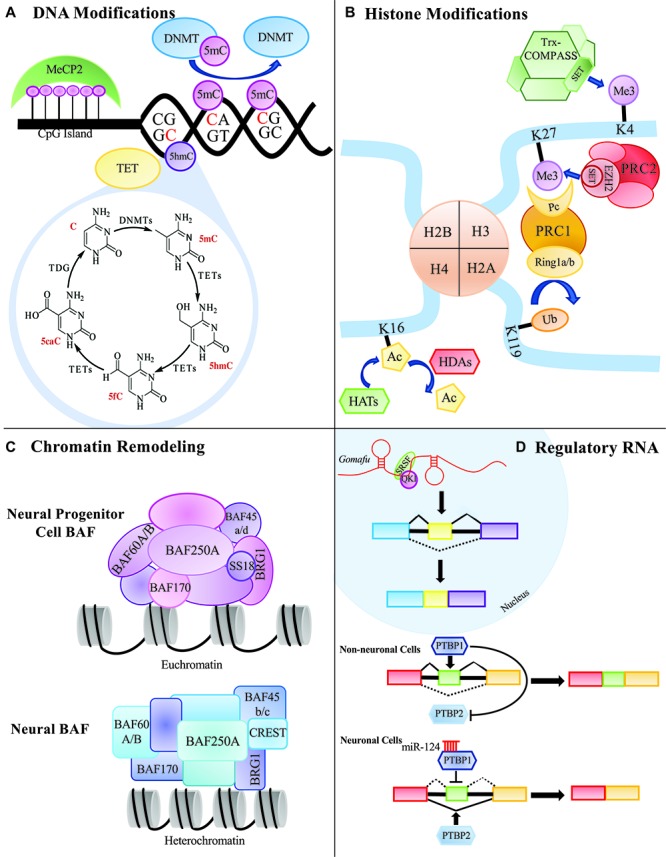
Summary of epigenetic processes that can occur in the mammalian central nervous system. **(A)** DNA modifying proteins can methylate CG of CH dinucleotides. Methylated cytosines can be further modified to 5hmC, 5fC, or 5caC to be replaced with an unmodified cytosine through thymine DNA glycosylase, TDG. **(B)** Histone modifiers can add various groups to the tails of the histone proteins that can affect the expressivity of a gene’s transcript. **(C)** Chromatin remodeling proteins can remodel the chromatin environment by affecting how tightly and loosely packed histones are and ultimately contribute to the gene’s expression. **(D)** Regulatory RNAs, such as long non-coding RNAs and microRNAs, can affect alternative splicing and protein expression.

The concept of epigenetics was first proposed in 1939 by Conrad Waddington to describe early embryonic development (Waddington, 1939). He proposed that development originates from the interactions of the starting material in the fertilized egg, and that the interactions give rise to something new. He further postulated that this process cycles, leading to the formation of a whole organism. Today, the accepted definition of epigenetics is the study of modifications that directly affect the expression of a gene, but do not change the underlying DNA sequence (Goldberg et al., 2007; Allis and Jenuwein, 2016; Gayon, 2016). There are several major epigenetic mechanisms that are extensively studied including DNA modifications, histone modifications, chromosome remodeling and RNA regulation via non-coding RNAs such as microRNA (miRNA) and long non-coding RNA (lncRNA) (Figure 1). Modifications can be added, removed and interpreted by various classes of proteins collectively known as ‘writers,’ ‘erasers’ and ‘readers,’ respectively. Disruption of these epigenetic mechanisms and their molecular machinery can have catastrophic consequences in the mammalian central nervous system (CNS).

Both nervous system development and function can be affected by epigenetic spatiotemporal regulation of gene expression. In the mammalian CNS, epigenetic dysregulation is associated with neuropsychiatric diseases such as major depressive disorder (MDD), autism spectrum disorders (ASDs), Fragile X, Rett syndrome and schizophrenia. Epigenetic studies are actively trying to identify biomarkers that could be associated with diseases to aid in our development of novel therapeutics. This information is critical, as the prevalence of neuropsychiatric diseases is on the rise (Atladottir et al., 2015). Here, we review the current understanding of epigenetic regulation in brain development and functions, with a focus on DNA methylation, as well as their implications in psychiatric diseases.

## DNA Methylation

### Functional Roles of DNA Methylation

DNA methylation is one of the best characterized epigenetic marks studied and has been regarded as a highly stable mark found in differentiated cells (Reik, 2007; Suzuki and Bird, 2008). It involves the covalent methylation of the fifth position in the cytosine ring, generating 5-methylcytosine (5mC) (Figure 1A). DNA methylation largely occurs at CpG dinucleotides (Bird, 1986). Accumulation of short, unmethylated CpG-rich clusters known as CpG islands occurs in the promoter regions of most genes (Jones, 2012). Genome-wide studies have implicated that the distribution of 5mC in transcripts could have differential roles in gene expression. For example, methylation status of the CpG islands helps to determine whether the corresponding gene will be expressed, whereas gene body methylation has been proposed to promote transcriptional elongation (Neri et al., 2017) and affect splicing (Maunakea et al., 2013). In addition, the methylation status of CpG islands can be influenced spatially based on tissue and cell type (Illingworth and Bird, 2009). For instance, the gene *HTR2A*, which has been implicated in many neuropsychiatric disorders (Norton and Owen, 2005), shows differential expression in the cerebellum and the cortex and is regulated by DNA methylation (Ladd-Acosta et al., 2007). Strikingly, the methylated CpG loci regulating *HTR2A* expression is over 1 Kb upstream of the promoter rather than being in the promoter region, illustrating that methylation can regulate genes across long distances. Thus, DNA methylation has important roles for brain region-specific transcriptome profiles.

Not only can DNA methylation regulate protein coding genes, it can also regulate non-protein coding RNA like lncRNAs. Random X-inactivation, an essential embryonic event, is triggered by the production of *Xist*, a lncRNA that coats the X chromosome destined to be inactivated (Borsani et al., 1991; Brown et al., 1992). The promoter of the *Xist* gene contains a CpG island whose methylation status ultimately dictates whether the X chromosome is active (Beard et al., 1995). How DNA methylation regulates lncRNA in the brain is still unclear. One study compared the DNA methylation patterns around the transcription start sites (TSSs) of protein coding genes and lncRNA loci (Sati et al., 2012). Surprisingly, a sharp increase in DNA methylation immediately downstream of the TSS was associated with lncRNA loci, but did not correlate with expression of the lncRNA. While this finding suggests that DNA methylation may not play an essential role in lncRNA expression, it would be interesting to investigate if blocking methylation at these sites influenced lncRNA expression.

In addition to its roles in gene regulation, DNA methylation also maintains genomic stability by controlling the expression of highly repetitive regions in the genome such as retrotransposons and satellite DNA (Liu et al., 1994; Woodcock et al., 1997; Walsh et al., 1998). In general, long interspersed nuclear element-1 (LINE 1) is only active in the germline and during early development (Ma et al., 2010). During somatic cell differentiation, DNA methylation silences LINE 1. Interestingly, studies have suggested that LINE 1 may be active during human and rodent neuronal differentiation and influence neuronal gene expression to create cell heterogeneity in the adult brain (Muotri et al., 2005; Muotri and Gage, 2006; Coufal et al., 2009). Indeed, LINE 1 has been shown to be more active in the brain compared to other tissues (Coufal et al., 2009). Increases in LINE 1 and other repetitive elements have been associated with the neuropsychiatric disorder Rett syndrome (Muotri et al., 2010). Suppression of LINE 1 requires methylation of its promoter and binding of the methyl-binding protein MeCP2, which plays a causal role in Rett syndrome.

Suppressing the expression of repetitive elements is one way by which DNA methylation maintains genomic stability and integrity. Genome instability has been shown to be highly correlated with many neuropsychiatric diseases such as schizophrenia, autism, Rett syndrome and several others (Smith et al., 2010). Numerous genes associated with these disorders, particularly schizophrenia and autisms, co-localize with regions of the genome that are more susceptible to mutations, or epigenetic alterations known as fragile sites. The most studied fragile site is associated with Fragile X syndrome and will be discussed later in this review.

Finally, DNA methylation has important roles in early developmental processes such as gene imprinting. Often, the “imprint” is methylation of a long-range control element called an imprint control element (ICE) (also referred to as imprint control region, ICR, or imprint center, IC) (Li et al., 1993; Barlow, 2011). Parental specific methylation of the ICE is established by the DNA methyltransferase (DNMT) complex DNMT3A/3L during gamete development (Bourc’his et al., 2001; Kaneda et al., 2004). Of the approximately 100 imprinted genes currently known, the majority of them are expressed in brain tissues, though not always exclusively, and have been reviewed previously (Wilkinson et al., 2007). One of the more extensively studied imprinted genes, specifically in the CNS of mammals, is the paternally expressed gene *Necdin* (*Ndn*) (Aizawa et al., 1992). *Ndn* regulates neuronal differentiation and axonal outgrowth. Also, *Ndn* is most highly expressed during mouse neuronal generation and between postnatal days 1–4.

### DNA Methylation in the Brain

DNA methylation in the brain is required for brain development and function throughout all stages in life. Dynamic regulation of DNA methylation is critical for cellular differentiation. One study compared the changes in DNA methylation patterns between two differentiation phases: the transition of embryonic stem cells (ESCs) to neuronal progenitor cells (NPCs), and the transition of NPCs to differentiated neurons (Mohn et al., 2008). The most dynamic changes in DNA methylation patterns were found when ESCs lost their pluripotency and became NPCs. In fact, ESCs were nearly devoid of DNA methylation marks except at the promoters of genes that were germline specific. In contrast, during the differentiation of NPCs to mature neurons, only 2.3% of the analyzed promoters gained *de novo* methylation and only 0.1% of promoters were demethylated, suggesting that the majority of DNA methylation dynamics do not occur in this phase. Similar to neurogenesis, astrocytogenesis is tightly controlled by DNA methylation. In mouse, astrocyte differentiation from neuroepithelial cells requires that the promoter of the *GFAP* gene be demethylated on embryonic day 14.5, allowing for the transcription factor STAT3 to bind and activate *GFAP* expression (Teter et al., 1994; Takizawa et al., 2001).

Very few studies have focused on how DNA methylation regulates other brain developmental features, such as neural migration and axonal/dendritic outgrowth. Two recent studies have demonstrated that the DNA methyltransferase, DNMT1, as having putative regulatory roles in immature GABAergic interneuron migration (Pensold et al., 2017; Symmank et al., 2018). They found that Dnmt1 promotes the migration and survival of immature migratory GABAergic interneurons that derive from the embryonic preoptic area (POA) by repressing *Pak6* expression (Pensold et al., 2017). p21-active kinases (PAKs) are known for their roles in cytoskeletal organization (Kumar et al., 2017), and *Pak6* has previously been shown to stimulate neurite outgrowth in post-migratory neurons derived from POA (Civiero et al., 2015; Pensold et al., 2017). *De novo* methylation by Dnmt3b in early embryonic neurodevelopmental processes has been shown to be critical in regulating the clustered protocadherins (*Pcdhs*) genes (Toyoda et al., 2014). Protocadherins are cell-surface adhesion proteins that are predominantly expressed in the nervous system (Sano et al., 1993), and have critical functions in neurite self-avoidance (Lefebvre et al., 2012), neuronal survival (Wang et al., 2002b), and dendritic patterning (Garrett et al., 2012). In mammals, they are found in three closely linked gene clusters call α (*Pcdha*), β (*Pcdhb*), and γ (*Pcdhg*) (Kohmura et al., 1998; Wu and Maniatis, 1999). Interestingly, the *Pcdhs* are stochastically expressed by alternative promoters in individual neurons generating single cell diversity of isoforms in the brain (Wang et al., 2002a). This stochastic expression is regulated by methylation of variable exons and this has been thoroughly reviewed elsewhere (Hirayama and Yagi, 2017). Protocadherins have critical roles in neural development and are starting to be implicated in neuropsychiatric disorders such as ASDs, depression and schizophrenia (Redies et al., 2012; El Hajj et al., 2017).

DNA methylation also has roles in brain function such as memory processing. In the mammalian brain, the hippocampus and the cortex are largely responsible for memory formation and storage (Morris et al., 1982; Squire, 1986; Miller et al., 2010). In the hippocampus, contextual fear conditioning induced changes in DNA methylation during memory formation in rats. When DNMTs were inhibited by either zebularine or 5-aza-2′-deoxycytidine, neuronal plasticity-promoting genes *Bdnf* and *Reelin* demonstrated altered methylation patterns (Levenson et al., 2006). After contextual fear conditioning, *Dnmt3a* and *Dnmt3b* mRNA were highly upregulated in the brain; however, when DNMT inhibitors, zebularine or 5-aza-2′-deoxycytidine, were injected into the hippocampus immediately after contextual fear conditioning, the fear response was eliminated, suggesting that DNA methylation is required for memory formation (Miller and Sweatt, 2007). Importantly, when the memory suppressor gene *Pp1* was examined after fear conditioning, there was an increase in methylation at the CpG island upstream of the *Pp1* transcriptional start site. It was postulated that the increase in *de novo* Dnmts may be necessary to transcriptionally silence memory suppressor genes after fear conditioning training to allow for memory formation and consolidation. In addition to the formation of memories, DNA methylation also has putative roles in long-term memory storage. Contextual fear conditioning was found to disrupt DNA methylation at three genes associated with memory, *Egr1*, *reelin*, and *calcineurin*, which also happen to have large promoter CpG islands (Miller et al., 2010). Both *reelin* and *calcineurin* were hypermethylated; however, only *calcineurin* maintained this hypermethylated state for 30 days, suggesting that DNA methylation might be required for long term memory storage.

Worth noting is that DNA methylation patterns in the brain can be affected by external stimuli in one’s environment. Interestingly, a study found that in mature neuronal cells, CpGs in low density regions compared to CpG islands undergo dynamic DNA methylation changes in response to electroconvulsive stimulation (Guo et al., 2011a). Numerous studies have shown that maternal care during childhood (Weaver et al., 2004), early life stressors including abuse (McGowan et al., 2009), parental separation and social defeat stressors can alter DNA methylation patterns in the brain and have been reviewed elsewhere (Yu et al., 2011).

### DNA Methyltransferases

**FIGURE 2:**
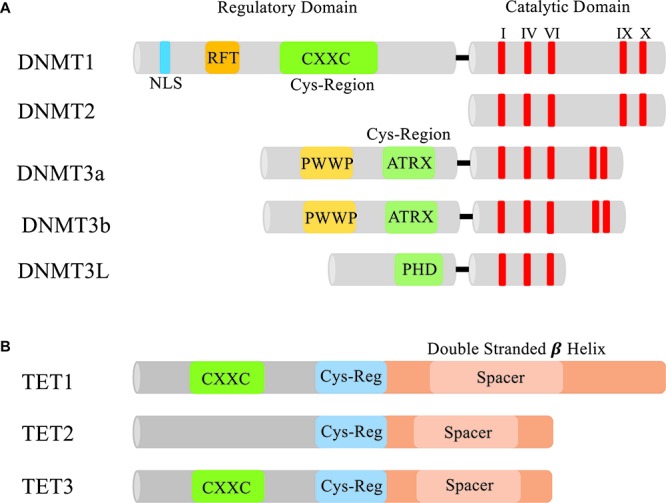
Domains of DNMTs and TETs. **(A)** The N-terminal and C-terminal domains of DNMTs. In the N-terminus of each Dnmt is a cysteine rich region. In Dnmt1 this region contains a CXXC zinc finger which is thought to aid in DNA binding (Frauer et al., 2011). Dnmt3a and 3b both contain a PWWP domain that specifically recognizes the repressive histone 3 lysine 36 trimethylation mark (H3K36me3) found in heterochromatin (Dhayalan et al., 2010). Dnmt3L contains a PHD-like cysteine rich domain that closely resembles the PHD domain encoded in Dnmt3a and 3b’s ATRX domain (Hata et al., 2002). All the DNMTs have a conserved C-terminal catalytic domain (I, IV, VI, IX, and X are the most conserved motifs in cytosine methyltransferases) responsible for modifying pyrimidines. NLS, nuclear localization signal; RFT, replication foci-targeting domain. **(B)** Domains of TET1, TET2, and TET3. Each TET protein has a core catalytic domain structured as a double stranded beta helix and a cys-regulatory region. Only TET1 and TET3 contain a CXXC domain to facilitate chromatin binding.

DNA methylation is generated by a group of DNMTs, also regarded as 5mC enzymatic “writers” (Figure 1A). Each Dnmt (Dnmt1, 3a, 3b, 2, and 3L) has evolved to have its own specialized regulatory functions. These specialized functions could be attributed to the lack of sequence homology seen in the N-terminal regulatory domains of the Dnmts (Bestor and Verdine, 1994). All of the Dnmts contain some version of a cysteine rich domain that further define their functions. The most conserved region between the DNMTs is the C-terminal catalytic domain, which is characteristic of all enzymes that modify pyrimidines at the fifth position (Figure 2A).

The first Dnmt purified was Dnmt1 back in 1983, and was found to be responsible for maintaining methylated CpG sites during DNA replication (Bestor and Ingram, 1983). Dnmt1 interacts with replication machinery, such as proliferating cell nuclear antigen (PCNA). Maintenance of the genomic methylation pattern requires that unmethylated regions also be maintained during replication. During the S phase, the transcription factor p21 blocks Dnmt1 from interacting with PCNA, which ensures that unmethylated regions maintain their original state (Chuang et al., 1997). This regulation of Dnmt1 plays an important role in asynchronous replication, specifically at replication origins that include CpG islands (Delgado et al., 1998). Mutation and loss-of-function studies have demonstrated the necessity of Dnmt1 during embryonic development. By gestational day 9.5, *Dnmt1*-null mouse embryos failed to develop and died by gestational day 11 (Li et al., 1992). In addition, overall global methylation levels decreased by threefold in the *Dnmt1*-null embryos.

Nearly 15 years later, two additional Dnmts were discovered, Dnmt3a and Dnmt3b. Both Dnmt3a and Dnmt3b are responsible for *de novo* methylation, which is also critical during early embryogenesis (Okano et al., 1998a). When either *Dnmt3a* or *Dnmt3b* are deleted during embryogenesis, severe developmental defects or embryonic lethality are observed, respectively (Okano et al., 1999). Mouse embryos with *Dnmt3a* depletion appear normal at birth, but die around 4 weeks of age. In contrast, embryos null for *Dnmt3b* were not viable and had growth retardation and neural tube defects. In addition to embryonic development, the *de novo* methyltransferases work in conjunction with Dnmt1 to regulate genome stability and imprinted genes. At a global level, deletion of *Dnmt3a* and/or *Dnmt3b* results in slight demethylation at repetitive sequences, but not to the same extent observed in *Dnmt1* gene deletion. This indicates that Dnmt1 is more important for the maintenance of methylation at repetitive sequences. At a loci-specific level, deletion of *Dnmt3a* and/or *Dnmt3b* has varied effects. For example, at several imprinted gene loci, *Igf2r* and *H19*, neither single nor dual gene disruption of *Dnmt3a* or *Dnmt3b* resulted in the demethylation pattern observed in *Dnmt1* gene disruption. However, at another imprinted loci, *Igf2*, dual deletion of *Dnmt3a/Dnmt3b* showed demethylation levels comparable to *Dnmt1* loss, whereas single gene disruption had no effect on demethylation. This indicates that there is some overlap in the roles of the Dnmts at certain gene sites.

Lesser known methyltransferases include Dnmt2 and Dnmt3L that were identified by sequence homology studies. Dnmt2 contains all of the C-terminal catalytic domains necessary to act as a methyltransferase; however, it was found to be non-essential for maintenance or *de novo* methylation (Okano et al., 1998b), but rather responsible for tRNA methylation (Goll et al., 2006; Schaefer et al., 2010). Dnmt3L demonstrates homology with Dnmt3a and Dnmt3b, but lacks the enzymatic activity required to generate *de novo* methylation (Bourc’his et al., 2001; Hata et al., 2002). Instead, Dnmt3L is essential in the establishment of maternal imprints and co-localizes with Dnmt3a/3b to regulate imprinting. Furthermore, in the male germ line, loss of Dnmt3L resulted in the reactivation of retrotransposons and meiotic failure in spermatocytes (Bourc’his and Bestor, 2004), suggesting a role in genomic stability.

### DNA Methyltransferases in the CNS

As writers of DNA methylation, Dnmts play critical roles in the mammalian CNS. Studies conducted on embryonic and adult mice revealed that Dnmts are highly expressed in neural progenitor cells, but are maintained at substantially lower levels in most differentiated neurons (Goto et al., 1994). Furthermore, mouse studies revealed that in the CNS, Dnmt3a is detected as early as embryonic day (E) E10.5 in the ventricular and subventricular zones, but its expression is predominantly in adult post-mitotic neurons (Feng et al., 2005). In contrast, Dnmt3b could only be detected during early neurogenesis. These specific time points of expression suggest that Dnmt3b may be important during the early stages of brain development, whereas Dnmt3a is more crucial to mature neurons. Further supporting different spatiotemporal roles for the *de novo* methyltransferases, it was shown that *Dnmt3b* is required for methylation at centromeric minor satellite repeats during embryonic brain development, whereas *Dnmt3a* is not (Okano et al., 1999).

Targeted mutagenesis studies revealed how critical the Dnmts are in the CNS. Conditional deletion of *Dnmt1* in CNS precursor cells, but not post-mitotic neurons, caused daughter cells to be severely hypomethylated (Fan et al., 2001). Interestingly, mice that had 30% of their CNS cells mutated showed selective pressure against the *Dnmt*-knockout cells in their brain. Three weeks after birth, all *Dnmt*-knockout cells were abolished. In adult forebrain neurons, double knockout of both *Dnmt1* and *Dnmt3a* (but neither gene by itself) resulted in significantly smaller hippocampi and dentate gyrus brain regions, due to smaller neurons (Feng et al., 2010). These mice also showed impairments in learning and memory as well as inappropriate upregulation of immune genes associated with demethylation. These results suggest that Dnmt1 and Dnmt3a may have redundant roles in post-mitotic neurons.

To further enhance the elaborate network of DNA methylation in the mammalian CNS, non-CpG dinucleotide methylation (CpH) has surfaced and shown to be highly enriched and have critical roles in the brain (Figure 1A). CpG dinucleotides make up around 75% of total cytosine methylation, whereas CpH dinucleotides (‘H’ could be adenosine, thymine or cytosine) make up the remaining 25% (Guo et al., 2014). Interestingly, CpH methylation is enriched in low CpG dense regions, is associated with repressed gene expression, but is unassociated with protein–DNA interaction sites. As previously mentioned, Dnmt1 preferentially associates with CpG dinucleotides, and maintains symmetric CpG methylation on both strands of DNA during replication. This symmetric balance is further facilitated by the complimentary base pairing (GpC). CpH methylation does not maintain the sequence symmetry and consequently during replication, CpH methylation is not conserved. This requires the re-establishment of CpH methylations after each cell division (Shirane et al., 2013). Re-establishment of CpH methylation has been linked to *Dnmt3a* gene expression (Xie et al., 2012; Shirane et al., 2013; Varley et al., 2013). In knockdown experiments, loss of *Dnmt3a*, but not *Dnmt1* or *Dnmt3b*, resulted in reduced CpH methylation with no effect on CpG methylation (Guo et al., 2014).

Like CpG methylation dynamics in early development, CpH methylation levels change during development. CpH methylation has been shown in relatively high abundance in stem cells (Lister et al., 2009; Laurent et al., 2010) and found to be enriched in both adult mouse and human brain tissues (Xie et al., 2012; Lister et al., 2013; Varley et al., 2013). A recent study showed that CpH methylation accumulates in the frontal cortex of the brain early after birth through adolescence and then slightly diminishes during aging (Lister et al., 2013). Different subclasses of neurons have unique CpH and CpG methylomes and CpH methylation may correlate more robustly with gene expression as compared to CpG methylation (Mo et al., 2015).

### Methyl-Binding Proteins

After the establishment of DNA methylation marks by “writers,’ a subset of proteins with methyl binding abilities known as “readers” can bind, protect and interpret these marks and facilitate function (Figure 1A). There are two main classes of methyl-CpG-binding proteins that have been thoroughly reviewed elsewhere (Ballestar and Wolffe, 2001), so this review will briefly discuss methyl-CpG-binding domain (MBD) proteins and MeCP2. Both protein families, for the most part, selectively bind to methylated DNA and aid in transcriptional repression (Hendrich and Bird, 1998). MeCP2 can facilitate gene repression by recruiting histone deacetylase (HDAC) machinery that further remodel the chromatin environment, facilitating a repressed state (Jones et al., 1998; Nan et al., 1998; Fuks et al., 2003). Later it was found that MeCP2 could also bind to non-CpG methylation modifications (Mellen et al., 2012; Guo et al., 2014; Gabel et al., 2015).

Methyl-binding proteins are ubiquitously expressed in somatic cells, but are particularly enriched in the mammalian CNS (Hendrich and Bird, 1998; Nan et al., 1998; Shahbazian et al., 2002; Cassel et al., 2004; Mullaney et al., 2004). Several studies have found that MeCP2 is involved in the regulation of brain-derived neurotrophic factor (*BDNF*), which promotes neuronal maturation (Chen et al., 2003; Martinowich et al., 2003). Additionally, MeCP2 was found to regulate a maternally imprinted gene called *Dlx5* that is part of the gamma-aminobutyric acid (GABA) pathway for inhibitory GABAergic neurons (Horike et al., 2005). Importantly, mutations in the MBD of MeCP2 have been implicated in the X-linked, neurodevelopment disorder known as Rett syndrome (Amir et al., 1999).

In addition to MeCP2, there are four other mammalian MBD proteins. MBD1-3 are known for their roles in transcriptional repression, whereas MBD4 functions as a thymine glycosylase in the mismatch repair pathway (Fujita et al., 2003). The MBD proteins can repress gene expression in several ways. One is through the recruitment of the H3K9 methyltransferase Suv39h1 and heterochromatin protein 1 (HP1). Both Suv39h1 and HP1 interact with MBD1 and aid in the establishment and maintenance of a repressive chromatin state which is further facilitated by the recruitment of both HDAC1 and HDAC2 (Fujita et al., 2003). During the S phase of DNA replication, regions of the chromosome that are repressed by DNA methylation, or histone modifications must be maintained. MBD1 forms an S phase specific complex with the H3K9 methyltransferase SETDB1, and then associates with chromatin assembly factor (CAF-1) to help maintain a repressed chromatin state (Sarraf and Stancheva, 2004). MBD2 and 3 were found to be in the nucleosome remodeling and histone deacetylation (NURD) complex, further associating the cross-talk of DNA methylation with histone modifications and chromatin remodeling enzymes (Zhang et al., 1999). Although MBD3 cannot bind methylated DNA, it was found to mediate the association between metastasis-associated protein 2 (MTA2), a MBD-containing protein, and the HDAC core of the NuRD complex. MBD2 is thought to direct the NuRD complex to methylated DNA and aid in the maintenance of a repressed environment.

Very little work has been done to identify functions of MBD1-3 in the CNS. Mice with a loss-of-function *MBD1* gene showed normal development, but as adults exhibited deficits in neurogenesis, impaired spatial learning and reduced long-term potentiation in the dentate gyrus (Zhao et al., 2003). Additionally, MBD1 was most enriched in hippocampus. During early embryogenesis, MBD3 was found to be highly expressed in the developing brain compared to MBD2 expression (Jung et al., 2003). In addition, in the adult brain, MBD3 is highly expressed in hippocampal and cortex neurons, but has very little expression in the outer cortical layer. Based on overall brain region enrichment patterning, it appears that the MBD proteins have some role in adult neurogenesis, but to what extent is unknown.

## DNA Demethylation

### Mechanism of DNA Demethylation

The mammalian genome undergoes genome-wide passive and active DNA demethylation processes during early embryogenesis and in the germline (Monk et al., 1987; Kafri et al., 1992; Tada et al., 1998). During passive demethylation, there is either a lack of, or inhibition of Dnmt1 preventing the replacement of methyl marks (Howlett and Reik, 1991; Mertineit et al., 1998; Rougier et al., 1998; Howell et al., 2001). Furthermore, Dnmt1 is unable to recognize and bind to unmethylated DNA (Valinluck and Sowers, 2007), rather it prefers to bind to hemi-methylated DNA. The precise molecular events of active DNA demethylation were not elucidated until 2009 when two seminal studies identified the presence of 5-hydroxymethylcytosine (5hmC) in the mammalian genome (Kriaucionis and Heintz, 2009; Tahiliani et al., 2009). Tahiliani et al. (2009) discovered that Ten-Eleven Translocation 1 (TET1) could oxidize the methyl group on 5mC to generate 5hmC (Figure 1A). Subsequent studies further identified TET2 and TET3 proteins as additional “erasers” of 5mC (Ito et al., 2010). 5hmC can be furthered catalyzed by all TETs to form 5-formylcytosine (5fC) and 5-carboxylcytosine (5caC) (He et al., 2011; Ito et al., 2011). In addition, 5hmC can be converted to 5-hydroxymethyluracil (5hmU) via the activation-induced cytidine deaminase (AID) and apolipoprotein B mRNA-editing catalytic polypeptides (APOBEC) enzymes (Bhutani et al., 2011). All three of these derivatives (5fC, 5caC, and 5hmU) can be cleaved by thymine-DNA glycosylase (TDG), which excises the modified cytosine base allowing for the base excision repair (BER) pathway to return it to an unmodified cytosine base (Bhutani et al., 2011; He et al., 2011). Contrary to previous belief that the accumulation of 5hmC was solely dependent on TET activity on 5mC, recent work has suggested that Dnmt1 and Dnmt3a, drive the initial accumulation of 5hmC in the early mouse zygote stage (Amouroux et al., 2016). Knockout models and small molecule inhibitor studies were able to uncouple the formation of 5hmC from 5mC in the paternal pronucleus. This suggests that 5hmC could itself be an independent epigenetic modification.

### TET Enzymes

TET enzymes catalytically oxidize the methyl group on 5mC to form 5hmC. The TET protein family is made up of three members: TET1, 2 and 3 (Figure 2B). Each contains a core catalytic domain structured as a double-stranded β-helix (DSBH) fold (Iyer et al., 2009; Tahiliani et al., 2009). Distinguishing the TET proteins from other related TET J-binding proteins (TET-JBP) families is the presence of a Cys domain located in the N-terminus of the DSBH domain that is thought to be essential for the catalytic activity. Also contained in TET1 and TET3 is a CXXC domain allowing the TET proteins to associate with chromatin through its binding to methylated cytosines. During development, the TET proteins can elect both an activating and repressive response from the genes they control based on what cofactors associate with them. In ES cells, TET1 has a repressive role when bound to the promoter region because it recruits MBD3-NURD (Yildirim et al., 2011) and SIN3A (Deplus et al., 2013). On the other hand, TET2 is not able to recruit either repressive component and has been associated with active cofactors such as Nanog and OGT (O-GlcNAc transferase) (Costa et al., 2013; Vella et al., 2013). In the male pronucleus, TET3 is responsible for the complete loss of 5mC and the accumulation of 5hmC, as shown by antibody staining and TET3 knockdown studies (Gu et al., 2011; Iqbal et al., 2011; Wossidlo et al., 2011).

### TET Enzymes in the CNS

Once it was discovered that TET enzymes were the long sought-after DNA demethylases, (Iyer et al., 2009; Tahiliani et al., 2009) extensive efforts were made to understand the dynamics of the global demethylation events observed in early embryogenesis. The catalytic function of the TET enzyme family and their putative novel roles were yet to be discovered. Even after all the advancements made in the past 9 years, very little is known about the function of TET enzymes in the mammalian CNS. Although all three TET proteins are expressed in the brain, Tet2 and Tet3 have higher expression compared to Tet1 (Kriaucionis and Heintz, 2009; Szulwach et al., 2011; Hahn et al., 2013). When Tet2 and Tet3 are overexpressed, premature neuronal differentiation was observed, whereas knockdown caused defects in differentiation progression (Hahn et al., 2013). *Tet1* knockout studies have identified several neural activity-regulated genes that are downregulated. Animals with this knockout display abnormal hippocampal synaptic plasticity and impaired memory extinction (Rudenko et al., 2013). Intriguingly, *Tet1* deletion did not appear to affect anxiety or depression related behaviors. Due to the embryonic lethality of *Tet3* deletion in mice, determining its function in the adult brain has been challenging. Instead of knockout studies, several groups have utilized small hairpin RNAs (shRNAs) to conditionally inhibit Tet3 expression. A recent study demonstrated that deletion of *Tet3*, and not *Tet1*, in mouse infralimbic prefrontal cortex (ILPFC), a region of the brain associated with fear extinction learning, impaired their ability to reverse a previously learned fear response (Li et al., 2014). Importantly, it was found that Tet3 mediates the drastic genome-wide redistribution of 5hmC in the ILPFC in response to extinction learning. Furthermore, posttraumatic stress disorders and phobias have been associated with impairments in fear extinction learning (Orsini and Maren, 2012).

### Roles of 5hmC, 5fC, and 5caC in the CNS

As previously discussed, 5hmC is the immediate product of TET enzymes’ in the demethylation of 5mC. Relative to other tissue types, 5hmC is found to be approximately 10 times higher in the brain compared to ESCs (Tahiliani et al., 2009; Globisch et al., 2010; Song et al., 2011). Genome-wide analysis studies have demonstrated that 5hmC is dynamically regulated in human (Wang et al., 2012) and mouse brains during neurodevelopment and aging (Szulwach et al., 2011). Dot blot analysis on cerebellum DNA showed 5hmC increased roughly 42% from fetal to adult brains. Furthermore, human 5hmC modifications were enriched at CpG islands and shores, exons and untranslated regions, consistent with 5hmC being associated with active genes. Notably, 5hmC has been found to be enriched at genes that are associated with ASDs. Differential hydroxymethylated regions found in human fetal and adult cerebellum were more likely to localize on Fragile X mental retardation protein (FMRP) target genes (Wang et al., 2012). These pieces of evidence clearly indicate the key roles of 5hmC in mammalian CNS. In addition to brain regions, some neurons have been found to contain high levels of 5hmC. For example, Purkinje neurons in the cerebellum were found to have roughly 40% more 5hmC relative to 5mC (Kriaucionis and Heintz, 2009). The enrichment of 5hmC in Purkinje neurons could account for its active biological functions as motor neurons that require an active transcriptome. Locus specific demethylation has been observed at the *Bdnf* loci. *Bdnf* is involved in adult neural plasticity and learning and memory (West et al., 2001). When cortical and hippocampal neurons experience a depolarization event, the *Bdnf* promoter is activated, enhancing its transcription (Shieh et al., 1998; Tao et al., 1998). The depolarization was also found to correlate with a decrease in CpG methylation in the *Bdnf* regulatory region (Martinowich et al., 2003; Guo et al., 2011b).

Very little is known about the functional roles of 5fC and 5caC other than their roles in active demethylation and conversion back to an unmodified cytosine. Genome-wide profiling studies found an enrichment of 5fC at poised and active enhancers, but with a clear preference for poised enhancers (Song et al., 2013). A recent study examined the dynamics of 5fC and 5caC in embryonic day 11.5 mice through 15-week-old adult mice (Bachman et al., 2015). They found that 5fC could be detected throughout all of the developmental time points, while 5caC could not be detected. Interestingly, both 5fC and 5caC were found to induce pausing of RNA Pol II during elongation, where this effect was not observed at C, 5mC nor 5hmC bases (Kellinger et al., 2012). It is possible that TDG could be recruited to sites of paused RNA Pol II to initiate the BER mechanism. Interestingly, TDG is the only glycosylase that is required for embryonic development (Cortazar et al., 2011; Cortellino et al., 2011). Even more intriguing is that in ESCs, both 5fC and 5caC recruit more proteins than either 5mC or 5hmC (Spruijt et al., 2013). The recruited proteins mostly had functional roles in DNA damage response (such as Tdg and p53), and proteins involved in chromatin remodeling (such as BAF170) were also found to interact with them.

## Histone Modifications

DNA is wrapped around a core histone octamer containing two copies each of the histone variants H2A, H2B, H3 and H4 forming a chromatin structure (Kornberg, 1974). The amino acids that make up the amino-terminal ‘histone tails,’ specifically lysines and arginines, are subject to modifications, such as methylation and acetylation, that can affect transcription (Figure 1B). Unlike DNA methylation which only has three major methyltransferases, there have been numerous histone methyltransferases and demethylases identified for histones (Hyun et al., 2017). The potential crosstalk between histone methylations and DNA modifications and chromatin remodelers and regulatory RNAs add another layer of complexity. These crosstalk events are thought to establish and maintain the local chromatin environment as well as help cells “remember” their differentiated state (Cedar and Bergman, 2009; Jobe et al., 2012). Several mechanisms facilitate this cross-talk such as DNMT3L and methyl-binding proteins like MeCP2 and MBD2, but we will focus in detail on the Polycomb (PcG) repressive proteins and the Trithorax (TrxG) activating proteins (Figure 1B). These two groups of proteins antagonistically regulate genes that are critical for development and cell differentiation pathways (Schwartz and Pirrotta, 2008). The proteins encoded by PcG and TrxG form large complexes to maintain the local chromatin environment in either a repressed or active state, respectively (Locke et al., 1988; Franke et al., 1992).

### Polycomb Group Proteins

The PcG proteins are divided into two major multiprotein complexes: polycomb repressive complexes 1 and 2 (PRC1 and PRC2) (Shao et al., 1999). Both complexes contain a core set of proteins critical for their basic function and can incorporate accessory proteins, permitting the complex to act in a spatiotemporal manner. There are four core proteins that are present in all PRC2 complexes: the SET domain contained in the enhancer of zeste [E(z), EZH1, and EZH2] protein, extra sex combs (Esc, EED) proteins, suppressor of zeste 12 [Su(z)12, SU(Z)12] and the histone binding protein p55 (RBAP48 and RBAP46) (Ng et al., 2000; Tie et al., 2001; Kuzmichev et al., 2002). The SET domain within E(Z) is responsible for the lysine methyltransferase activity specifically occurring on histone 3 at lysine 27 (H3K27) (Cao et al., 2002). PRC1 is also composed of a set of four major core proteins including polycomb (Pc), polyhomeotic (Ph), posterior sex combs (Psc) and Sex combs extra (Sce/dRing 1) (Shao et al., 1999). The chromodomain in Pc is responsible for recognizing and binding trimethylated H3K27 (H3K27me3) and upon binding will induce structural changes in the chromatin (Fischle et al., 2003; Min et al., 2003). In addition, PRC1 is also responsible for the monoubiquitination of lysines on histone H2A via the proteins Ring1A/B (de Napoles et al., 2004).

### Trithorax Group Proteins

Antagonistic to the PcG proteins, the TrxG proteins are recognized for their activating mechanisms and addition of histone 3 lysine 4 trimethylation (H3K4me3). TrxG proteins are also evolutionarily conserved and are categorized into three groups based on their function. Group one is composed of the SET-domain-containing proteins that methylate histone tails, group two contains ATP-dependent chromatin remodeling proteins and finally group three contains the TrxG proteins that can bind DNA in a sequence specific manner. Each of these groups are thoroughly reviewed elsewhere (Schuettengruber et al., 2011). One of the first SET-domain-containing histone modifying complexes identified that could catalyze mono-, di-, and trimethylation on H3K4 was a complex called COMPASS in yeast (Miller et al., 2001; Roguev et al., 2001). Mammals have six COMPASS-like complexes that have been shown to facilitate most H3K4me3 present, indicating that they are likely involved in global gene activation (Wu et al., 2008).

### PcG and TrxG Proteins in the CNS

In the mammalian CNS, both PcG and TrxG proteins help to regulate the differentiation process of neuronal cells. In ESCs, polycomb proteins prevent neuronal differentiation by adding H3K27me3 repressive marks at neuronal specific genes such as *Ngns, Pax6, Sox1* (Bernstein et al., 2006; Mikkelsen et al., 2007). However, these genes simultaneously contain the active trithorax H3K4me3 mark, making these promoters bivalent. As ESCs differentiate into NPCs, the H3K27me3 polycomb mark is removed specifically by the histone demethylase Jmjd3 to further commit them to a neural lineage (Burgold et al., 2008). In addition to histone demethylation, activation of the TrxG COMPASS-like complex proteins RBBP5 and DBY30 are essential for the differentiation of ESCs into NPCs (Jiang et al., 2011). In NPCs, the PRC2 subunit Ezh2 is initially highly expressed, but declines during cortical neuron differentiation (Pereira et al., 2010). The loss of Ezh2 was shown to augment neurogenesis and neuronal differentiation. PcG complexes have also been associated with differentiation of NPCs to astrocytes (Hirabayashi et al., 2009) and oligodendrocytes (Sher et al., 2008). As the brain develops, NPCs can travel up and outward to form the outer layers of the brain. A study demonstrated that Ezh2 silences genes associated with neuron migration, such as *Netrin1*, to maintain correct migration patterns throughout the brain (Di Meglio et al., 2013).

Furthermore, several studies have demonstrated the importance of cross-talk between DNA methylation and histone modifications during mammalian brain development (Wu et al., 2010a; Hahn et al., 2013). As previously described, during neurogenesis as NPCs begin to differentiate, there is an increase in 5hmC specifically in gene bodies of developmentally active genes with little change in 5mC. Accompanying this increase, there is also a decrease in Polycomb-mediated repression and H3K27me3 formation (Hahn et al., 2013). Overexpression of *Tet2* and *Tet3*, both of which are highly expressed in the embryonic cortex, prompted early differentiation of NPCs. An analogous and more obvious transition was seen when *Ezh2* was also depleted. Moreover, when Tet proteins were inhibited and *Ezh2* overexpressed, NPCs failed to differentiate. This suggests that Polycomb may regulate the transition of NPCs differentiation, and Tet proteins putatively maintain the differentiated state. Additionally, it has been demonstrated that there is an inverse association of Dnmt3a *de novo* methylation on non-promoter CpGs and H3K27me3 formation in the mouse brain (Wu et al., 2010a). Mice deficient for *Dnmt3a* had an increase of H3K27me3 as well as increases of PRC2 components Suz12 and Ezh2 at Dnmt3a targets. As previously discussed, Dnmt3a has more of a role in DNA methylation maintenance in postnatal development. The proposed cross-talk suggests that in addition to methylating promoters of self-renewal genes in NPCs, Dnmt3a also has an activating function by inducing transcription of mature neural genes by down regulating H3K27me3 and antagonizing PRC2 binding.

### Histone Acetylation

Methylation is just one type of modification that can be present on histone tails; acetylation is a second type of modification that also regulates chromatin dynamics. Histone acetyltransferases (HATs) and HDACs are enzymatic proteins that either add or remove acetylation residues on lysines, respectively (Inoue and Fujimoto, 1970; Racey and Byvoet, 1971) (Figure 1B). Core histones are acetylated by transcriptional coactivators like CBP/p300 that are ubiquitously expressed and involved in cell cycle control, differentiation and apoptosis (Yang et al., 1996). HATs can be divided into three families based on the structure of their catalytic domains: GNAT, MYST and CBP/p300 which are reviewed elsewhere (Sterner and Berger, 2000; Kouzarides, 2007). Supportive of their activating role, HATs will interact with various transcription factors to promote many signaling cascades (Saha and Pahan, 2006). Similar to methylation, acetylation is reversible and removed by HDACs that silence gene expression. HDACs can also be categorized into four distinct classes where class 1 and class 2 HDACs seem to have important roles in the nervous system (Gray and Ekstrom, 2001; Abel and Zukin, 2008). Inhibitors of HDACs have shown promising effects in treating both neurodegenerative and neuropsychiatric diseases. It has been demonstrated that HDAC inhibitors could re-establish histone acetylation that is potentially lost due to dysregulation of the HAT, Tip60 (Cao and Sudhof, 2001). Furthermore, inhibition of HDACs restored learning and memory in a mouse model of neurodegeneration (Fischer et al., 2007). In Fragile X studies, combined administration of 5-azadeoxycytidine and various HDAC inhibitors cause reactivation of *FMR1* gene expression (Chiurazzi et al., 1998). In the mouse brain, *Hdac3* deletion provoked abnormal locomotor coordination, sociability and cognition (Nott et al., 2016). Interestingly, a cross-talk between HDAC3 and MeCP2 was shown to positively regulate neuronal genes by deacetylating FOXO, a transcription factor that is highly expressed in the hippocampus. A putative link for this cross-talk in relation to Rett syndrome is discussed below.

## Chromatin Remodeling

The total length of DNA in one mammalian cell is on average 2 meters, yet the size of the nucleus is only 6 μm. In order to fit the entire genome into such a limited space, DNA molecules have to undergo extraordinary consolidation by a process termed chromatin remodeling. In addition to histones, a major contributor to chromatin compaction is a family of ATP-dependent remodeling proteins. The BAF (mammalian SWI/SNF) complex is a chromatin remodeling multiplex that uses ATP-dependent energy to modify the chromatin landscape to promote cell differentiation (Son and Crabtree, 2014) (Figure 1C). BAF complexes exist in a very spatiotemporal specific fashion. For example, in the mammalian CNS, there are developmental stage-specific BAF complexes in ESCs (Kaeser et al., 2008), NPCs and in post-mitotic neurons (Lessard et al., 2007). A unique feature to BAF complexes is that the alternative subunits that make up the various stage-specific complexes are not interchangeable, indicating their functions are non-overlapping (Wang et al., 1996a,b). Interestingly, BAF complexes are being increasingly associated with neuropsychiatric diseases such as ASD (Neale et al., 2012; O’Roak et al., 2012) and schizophrenia (Koga et al., 2009).

### BAF Chromatin Remodelers

The ESC specific BAF (esBAF) contains the ATPase BRG1, BAF250a, BAF60a/b and BAF155 (Kaeser et al., 2008). Deletion of any of the core subunits results in a lethal phenotype (Bultman et al., 2000). For example, shRNA depletion of *Brg1* impairs self-renewal properties of ESCs and results in loss of key ESC markers such as *Oct4*, *Sox2* and *Nanog* (Ho et al., 2009). In addition, deletion of *Brg1* also resulted in an increase of the PRC2 recruitment and subsequently, H3K27me3 repression at active ESC genes (Ho et al., 2011). All this evidence suggests that esBAF maintains a euchromatic environment that is required to maintain the pluripotency of ESCs.

The transition from esBAF to neural progenitor BAF (npBAF) is associated with the replacement of esBAF155 with npBAF170 (Ho et al., 2009; Tuoc et al., 2013). npBAF is composed of a combination of either ATPase BRG1 or BRM along with several other BAF subunits. Similar to esBAF, npBAF are critical for the self-renewal properties of NPCs and loss of *Brg1* shows similar phenotypes as those seen in esBAF. Interestingly, BAF170 was shown to interact with the transcription factor Pax6 whose primary function is to regulate neural progenitor division during early cortical development (Gotz et al., 1998). Upon BAF170 binding to Pax6, the transcriptional repressor REST (RE1-silencing transcription factor, also known as NRSF) is recruited, and represses Pax6 in non-neuronal radial glia cells (Tuoc et al., 2013). A conserved, 23 base pair sequence known as RE1 (repressor element 1, also known as NRSE) acts as the binding site for REST (Chong et al., 1995; Schoenherr and Anderson, 1995; Chen et al., 1998). Two corepressors are required for REST mediated silencing, Sin3-HDAC and the CoREST protein complex that contains HDACs (Andres et al., 1999; Grimes et al., 2000). Additionally, it was shown that CoREST interacts with BAF57, a subunit present in all stage-specific complexes, to induce long term silencing (Battaglioli et al., 2002). BAF170 is present in the subset of radial glia cells that are destined to be non-neuronal, and absent in radial glia cells destined to become intermediate progenitors that migrate outward to form the outer cortex layer (Andres et al., 1999; Grimes et al., 2000; Tuoc et al., 2013).

The substitutions of BAF53a for BAF53b, SS18 for CREST and BAF45a/d for BAF45b/c marks the transition from npBAF to the mature neuron (nBAF) complex (Olave et al., 2002). Importantly, the nBAF subunits are exclusive to neuronal cells and maintain the chromatin environment of post-mitotic neurons (Olave et al., 2002; Naik et al., 2007). nBAF, in complex with CREST, is essential in regulating dendritic outgrowth (Wu et al., 2007). Normal brain function depends on the correct wiring and synaptic function controlled by adequate dendritic outgrowth. Calcium regulation in the CNS can activate calcium mediated transcription factors, such as CREST, to promote the activation of genes required for dendrite growth (Aizawa et al., 2004).

## Regulatory RNA

An emerging field in epigenetics is focusing on debunking the large amount of non-protein coding DNA contained in the mammalian genome. Over the past 20 years, scientists have begun to discover that non-coding is not equivalent to non-functional. When transcribed, these regions generate non-coding RNA (ncRNA) that can range in size from just ∼21 nucleotides to 100,000 nucleotides and can post-transcriptionally regulate mRNA. Many flavors of ncRNAs have been identified (Cech and Steitz, 2014); however, this review will briefly cover miRNA and lncRNA and the putative functions they may serve in the mammalian CNS.

### MicroRNAs

MicroRNAs are roughly 22 nucleotides in length and have major roles in post-transcriptionally regulating gene expression by destabilizing their target mRNA (Bartel, 2004). Partial sequence complementarity to the 3′ untranslated region (3′UTR) of the target is adequate for gene downregulation (Lewis et al., 2005). Perfect complementarity is required at what is called the “seed sequence” in the 5′UTR of the miRNA. Interestingly, a single miRNA can target hundreds of different mRNA and that a single mRNA can be targeted by more than one miRNA (Lim et al., 2005). Determining functional roles for the hundreds of miRNAs discovered has eluded scientists for years. Early studies proposed that miRNA had extensive roles during mammalian brain development and several of these studies identified neural-specific miRNA (Krichevsky et al., 2003; Kim et al., 2004; Miska et al., 2004; Sempere et al., 2004). Of the neural-specific miRNA identified, one in particular stands out, miR-124. miR-124 is the most abundant and highly conserved miRNA found in the mammalian brain (Lagos-Quintana et al., 2002). Accounting for nearly 25–48% of all the miRNA in the brain, miR-124 has been implicated as a major contributor in neuronal differentiation and maturation (Krichevsky et al., 2006; Makeyev et al., 2007). For example, the direct targeting and repression of the RNA binding protein, PTBP1 by miR-124 has critical roles in non-neuronal cell development (Makeyev et al., 2007) (Figure 1D). PTBP1 is highly expressed in non-neuronal cells and inhibits alternative splicing of neuron-specific genes (Wagner and Garcia-Blanco, 2001; Sharma et al., 2005). In cells destined to become neurons, miR-124 binds and represses PTBP1, resulting in an increase of PTBP1’s neuronal homolog, PTBP2 protein expression, inducing neuron-specific alternative splicing.

Another brain enriched miRNA, miR-137, is thought to have roles in both adult neurogenesis and neuronal maturation. During adult neurogenesis, miR-137 regulation of proliferation versus differentiation is coupled with its ability to cross-talk with MeCP2 and Ezh2 (Szulwach et al., 2010). Roughly 2–4 Kb upstream of miR-137, methylated CpGs were found as well as a threefold enrichment of MeCP2 binding. Subsequently, it was found that Sox2 also binds upstream of miR-137, and concurrent binding of Sox2 with MeCP2 inhibited miR-137. When miR-137 expression is reduced, there is an increase in neuronal differentiation and a decrease in adult neural stem cell proliferation. This is concurrent with a previous observation that miR-137 expression increases during neuronal differentiation (Silber et al., 2008). The polycomb protein Ezh2, was found to be a direct target of miR-137 *in vitro* (Szulwach et al., 2010). MiR-137 reduces the expression of *Ezh2* and consequently there is also a decrease in H3K27me3. Loss of H3K27me3 encourages adult stem cells to begin to differentiate rather than proliferate. With regards to neuropsychiatric disorders, Genome wide association studies (GWAS) identified miR-137 as one of the strongest associated factors with schizophrenia (Schizophrenia Schizophrenia Psychiatric Genome-Wide Association Study (GWAS) Consortium, 2011; Kwon et al., 2013; Schizophrenia Working Group of the Psychiatric Genomics Consortium, 2014). Intriguingly, four targets of miR-137 were also found to be highly associated with schizophrenia (Kwon et al., 2013); however, the biological impact of miR-137 in schizophrenia still remains to be explored.

### Long Non-coding RNAs

Long non-coding RNAs are classified as having at least 200 nucleotides and non-protein coding abilities (Kapranov et al., 2007). They are also one of the least well understood class of ncNRAs because of the difficulty in distinguishing them from transcription by-products. Compositionally, lncRNA do not appear to be very well conserved between mouse and human (Pang et al., 2006). In the mouse genome, the vast majority of lncRNAs do not contain an open reading frame (Ravasi et al., 2006). In addition, compared to protein coding transcripts, lncRNA tend to be shorter and contain fewer introns. Unusually, some lncRNA such as the paternally imprinted lncRNA *H19*, are polyadenylated, spliced and exported to the cytoplasm just like protein coding transcripts (Brannan et al., 1990). Functional roles of lncRNA may depend on where in the genome they are located. Those that are transcribed near expressed genes have the potential to regulate the expression of that gene in *cis*. One of the most well studied lncRNAs is *Xist*, which functions in *cis* and is critical for inactivating one of the X chromosomes in mammalian females (Brockdorff et al., 1991). As *Xist* coats the X chromosome, other repressive factors are recruited, such as Polycomb repressive complexes PRC1 and PRC2 and other histone modifying enzymes (Plath et al., 2003; Silva et al., 2003; de Napoles et al., 2004). lncRNAs have also been demonstrated to regulate transcriptional repressors and activators from a distance (in *trans*). The *HOTAIR* lncRNA is 2.2 Kb in length, and was shown to repress the transcription of 40 Kb of the *HOXD* locus (Rinn et al., 2007). It is proposed that *HOTAIR* interacts with PRC2 to facilitate H3K27me3 of the *HOXD* locus because siRNA mediated knockdown of *HOTAIR* resulted in the loss of H3K27me3 marks specifically at *HOXD*. Beyond chromatin remodeling, other putative functions for lncRNAs have been suggested, such as transcriptional control and post-transcriptional processing, which are reviewed in detail elsewhere (Mercer et al., 2009; Ponting et al., 2009).

The role of lncRNAs in chromatin remodeling has been extensively studied and the scientific community is just starting to make strides in investigating their roles in the brain (reviewed by Ng et al., 2013). lncRNAs have been found in many tissues (Iyer et al., 2015), but are strikingly enriched in the mammalian brain. One study identified over 800 lncRNAs in the mouse brain, and found that most were associated with specific brain regions, cell types or subcellular compartments, suggesting some putative function (Mercer et al., 2008). One of the better studied lncRNAs in the brain is *Malat1* (also known as *NEAT2*), which is particularly enriched in neurons (Bernard et al., 2010; Lipovich et al., 2012). *Malat1* localizes to nuclear speckles which are storage/assembly sites for processing factors involved in pre-mRNA splicing (Lamond and Spector, 2003; Hutchinson et al., 2007; Clemson et al., 2009). Studies demonstrated that *Malat1* recruits SR splicing factors in the nuclear spectacle and can regulate genes involved in neural processes and synaptic function (Bernard et al., 2010). Importantly, *Malat1* was shown to have 90% conservation between human and mouse, suggesting maintenance of a critical function. A recent computational study utilized RNA-seq data from mouse embryonic brains to identify temporally regulated lncRNAs in brain development. Interestingly, lncRNAs specifically expressed in embryonic brains were no longer expressed in adult brains (Lv et al., 2013). Another study employed RNA-seq on human iPSCs to investigate the expression of lncRNAs during their differentiation into mature neurons (Lin et al., 2011). Several of the lncRNAs that were aberrantly regulated during differentiation were associated with candidate genes of neuropsychiatric disorders, such as ASDs, bipolar disorder and schizophrenia. Much research is being conducted on identifying and determining functional roles of the ever-growing list of lncRNAs; however, more work remains to be done.

To add another layer of complexity, different groups of non-coding RNAs have been found to cross-talk with each other and form regulatory networks in the brain (Kleaveland et al., 2018). A recent study found that in mouse brain, the lncRNA *Cyrano* destabilizes miR-7 through its highly complementary site for miR-7. Degradation of miR-7 promoted the accumulation of a circular RNA Cdr1as, which is known to dampen neuronal activity (Memczak et al., 2013; Piwecka et al., 2017). Interestingly, Cdr1as contains an inherent destruction mechanism where binding of miR-671 induces its slicing (Kleaveland et al., 2018). It has been proposed that because the binding sites for miR-7 and miR-671 are so close on Cdr1as, cooperative binding could recruit a silencing complex and control the accumulation of Cdr1as in the brain (Grimson et al., 2007; Saetrom et al., 2007).

Proper epigenetic regulations are critical for normal brain development and functions. Numerous evidences suggest that their dysregulation could serve as causal roles in the onset of neurological, neurodegenerative and neuropsychiatric disorders. In the following sections, we will focus on several neuropsychiatric disorders with known roles of epigenetic regulation in their etiology and progression (Table 1).

**Table 1:** Summary table of epigenetic processes that can occur in various neuropsychiatric diseases.

Disease	Epigenetic modifications	Description	Reference
Major depressive disorder	DNA modifications	• Hypermethylation at *BDNF* locus • CpG methylation in exon 1 biomarker for depressed patients • Patients may not respond to antidepressants • Antidepressants phosphorylate MeCP2 causing it to disassociate from methylated DNA •*SLC6A4* methylation may be related to depression heritability • Environmental stressors disrupt methylation • Child-hood stressors associated with increased methylation at exon 1F of *NR3C1* • Early life adversity changes methylation of CpG sites in *FKBP5* • Pcdh genes show reduced promoter methylation in response to good maternal care	Angelucci et al., 2005; Fuchikami et al., 2011 Tadic et al., 2014 Hutchinson et al., 2012 Mendonca et al., 2019 Farrell et al., 2018; Efstathopoulos et al., 2018 Farrell et al., 2018 McGowan et al., 2011
	Histone modifications	• HDAC2 reduced in nucleus accumbens and postmortem brains •*Hdac5* knockout (mouse) • Sensitive to depressive like behaviors • Hdac5 increased in hippocampus of chronically stressed mice • Hdac5 levels reversed with antidepressant treatment • HDAC inhibitors used as antidepressants • MS-275 reverses depressive behaviors	Covington et al., 2009 Renthal et al., 2007 Tsankova et al., 2006 Eckschlager et al., 2017;Covington et al., 2011
	Regulatory RNA	• miR-132 and miR-124 regulate BDNF • miR-132 potential biomarker for MDD • miR-124 not a reliable biomarker • miR-132 consistently identified in MDD studies • SSRI treatment increases miR-16 expression and regulates SERT uptake of serotonin • Patients with low miR-1202 levels predicted to respond better to serotonin-based drugs	Fang et al., 2018 Bocchio-Chiavetto et al., 2013; He et al., 2016 Baudry et al., 2010; [Bibr B389][Bibr B219];Fiori et al., 2017
Autism spectrum disorders	DNA modifications	• Hypermethylation of CpG islands at SHANK3	Zhu et al., 2014
	Histone modifications	• AUTS2 protein functioning in complex with PRC1 to promote gene activation • AUTS2 knockout (mouse) • Impaired developmental phenotypes seen in humans	Gao et al., 2012, 2014 Gao et al., 2014
	Regulatory RNA	• Found 28 differentially expressed miRNAs in cortex of ASD patients • Over 200 differentially expressed lncRNA in ASD brains	Abu-Elneel et al., 2008 Ziats and Rennert, 2013
Fragile X	DNA modifications	• Loss of *FMR1* results in hypermethylation of CGG repeat	Bell et al., 1991; Pieretti et al., 1991; Sutcliffe et al., 1992; Orsini and Maren, 2012
	Regulatory RNA	• Mutated *FMR1* associates with the RNA interference pathway	Jin et al., 2004a
Rett syndrome	DNA modifications	• Missense mutation in *MeCP2* •*Mecp2* knockout (mouse) • Impaired motor coordination; increase in anxiety; abnormal social behavior • Genes that acquire mCH more likely to be dysregulated in RTT mouse model •*MeCP2* duplication syndrome • Hypersynchrony in hippocampal neurons	Mellen et al., 2012; Lyst et al., 2013 Gemelli et al., 2006 Chen et al., 2015 Van Esch et al., 2005 Lu et al., 2016
	Histone modifications	• Lose MeCP2 interaction with NCoR/HDAC3 • Conditional knockout of *Hdac3* (mouse) • Lose Hdac3 and FOXO at promoters	Nan et al., 1998; Ebert et al., 2013; Lyst et al., 2013 Nott et al., 2016
	Regulatory RNA	• Downregulation of miR-146a and miR-146b in *Mecp2*-null mouse putatively upregulated Irak1 in RTT brain • *Bdnf* gene contains multiple miRNA binding sites	Taganov et al., 2006; Urdinguio et al., 2008; Nahid et al., 2009 Wu et al., 2010b
Schizophrenia	DNA modifications	• 50% increase in DNA methylation at *RELN* promoter • Transcription factor Sp1 signals for demethylation and prevents *de novo* methylation, possibly regulates *reelin* • Olanzapine treatment alters methylation at *Pcdha11*, *Pcdha9*, and *Pcdhga5*	Impagnatiello et al., 1998 Han et al., 2001; Chen et al., 2002 Melka et al., 2014
	Regulatory RNA	• miR-132 is downregulated and is associated with cognitive and behavioral impairments • miR-132 targets Dnmt3a • miR-195 upregulated and targets *BDNF, RELN* and *DRD1* • High risk SNP in miR-137 is most common among SZ patients • Earlier age of onset, abnormal neurodevelopment • lncRNA *GOMAFU* is reduced in SZ patients • QKI and SRSF interact with *GOMAFU* to regulate alternative splicing • Disruption of QKI could account for decreased myelin-related genes expression	Moreau et al., 2011; Miller et al., 2012 Cannon, 1996 Beveridge et al., 2010 Hamshere et al., 2013; Guan et al., 2014 Lett et al., 2013; van Erp et al., 2014 Barry et al., 2014 Aberg et al., 2006a


## Major Depressive Disorder

Individuals with MDD present clinically with not only a depressed mood, but can also suffer from anhedonia, dysregulated appetite and sleep, fatigue, poor concentration and suicidal ideations or acts (Belmaker and Agam, 2008). In the United States, the incidence of depression in women is greater than 20%, nearly twice that of men (Kessler et al., 2003). Twin studies have suggested that MDD has a high heritability rate of about 37% (Sullivan et al., 2000). However, MDD is not monogenic, but rather caused by many genes each contributing only a small proportion. Environment, such as early life stress or trauma, is a major risk factor. Many studies have tried to identify biomarkers to assess a patient’s predisposition for MDD; however, no useful biomarkers have yet been identified. Furthermore, many individuals with MDD are resistant to treatments, and so developing a greater understanding of the neurological facets of MDD has become paramount to the creation of efficacious therapies.

### Differential DNA Methylation

A major candidate gene for MDD is *BDNF*. Individuals with MDD show reduced BDNF protein, and multiple studies have associated this reduction with increased methylation of the *BDNF* promoter in peripheral blood cells (Angelucci et al., 2005). *BDNF* has two small CpG islands upstream of exons 1 and 4. One study found that the methylation status of exon 1 in *BDNF* could be used to accurately distinguish between MDD patients and healthy controls. Remarkably, the depressed patients consistently showed a complete absence of methylation at certain CpG sites in exon 1 (Fuchikami et al., 2011). Although this study was only based on a small number of participants, it would be worth investigating whether these findings could be replicated in larger populations. Absence of methylation at one particular CpG site in exon 4 of *BDNF* has been associated with reduced response to antidepressant drugs (Tadic et al., 2014). While antidepressants showed no effect on exon 4 methylation, *in vitro* experiments established that antidepressants could regulate the promoter activity of *BDNF*. Furthermore, antidepressants have been shown to increase *BDNF* expression in mice by phosphorylation of MeCP2, which causes the removal of MeCP2 from the DNA (Hutchinson et al., 2012). *BDNF* exon 4 methylation levels and circulating *BDNF* protein together may predict a patient’s treatment response (Lieb et al., 2018). These findings collectively suggest that *BDNF* methylation levels may be a useful biomarker and tool to make more informed choices about individual therapies. Another well-studied biological factor in MDD is the serotonin transporter gene *SLC6A4*. *SLC6A4* methylation correlates with depression in a variety of ways. For example, in an analysis of individuals with MDD, those who had a family member with depression showed a higher percentage of *SLC6A4* methylation, indicating that epigenetic regulation of this loci may be related to depression heritability. In mother–child pairs that were concordant for depression, increased methylation of the *SLC6A4* promoter was seen in both mother and child (Mendonca et al., 2019).

### Disruption of DNA Methylation From Environmental Stressors

Stressful, traumatic events in early life are a major environmental risk factor for MDD, and changes in stress-related genes may be part of the mechanism of depression for some individuals. The glucocorticoid receptor gene, *NR3C1*, plays an important role in the hypothalamic–pituitary–adrenal (HPA) axis, a stress response system that becomes dysregulated in MDD. Exon 1F of *NR3C1* has been extensively studied with regards to its role in early life adversity (Daskalakis and Yehuda, 2014), and has been the target of focus for many depression studies as well. Individuals with MDD show hypermethylation of *NR3C1* exon 1F, which correlated with morning cortisol levels (Farrell et al., 2018). In adolescent males, increased *NR3C1* exon 1F methylation was associated with stressful experiences such as being bullied, lacking friends and internalizing symptoms, as assessed by a depression scale (Efstathopoulos et al., 2018). Polymorphisms of the glucocorticoid receptor co-chaperone protein, FK506 binding protein 5 (*FKBP5*), have also been associated with MDD. Interestingly, methylation of certain CpG sites of *FKBP5* intron 7 significantly correlated with early life adversity in MDD patients (Farrell et al., 2018). Thus, the connection between many MDD cases and early life trauma involves disruption to the stress response system at an epigenetic level. It is plausible to imagine potential pharmacotherapies that could target methylation of key genes in this system to help restore balance in the HPA axis, and thus attenuate MDD symptoms. Whether targeting HPA axis genes alone would be enough to improve MDD, remains to be understood.

Several studies have linked clustered *Pcdhs* to depression-like behaviors. A rat model of depression revealed that *Pcdhga11* expression levels were increased in the hippocampus (Garafola and Henn, 2014), suggesting *Pcdhga11* could be used as a putative biomarker. In contrast to early life stressors, which epigenetically alter the HPA axis, positive early-life parental interactions can epigenetically alter genes that promote neuronal function. For example, adult mice that receive good maternal care (high licking), showed increased histone acetylation and DNA methylation in exons of *Pcdh* genes. Also, there was reduced methylation at their promoter, increasing over all expression of *Pcdh* genes (McGowan et al., 2011).

### HDAC Inhibitors as a Putative Antidepressant

Histone deacetylases are a promising target for MDD therapies. Mouse behavioral paradigms, such as chronic social defeat stress, have been relied upon as a way to measure antidepressant efficacy (Yin et al., 2016). In mice that have experienced chronic social defeat stress and in postmortem brains from humans with clinical depression, HDAC2 protein is reduced in the nucleus accumbens (NAc; a brain region associated with reward) (Covington et al., 2009). *Hdac5* expression is also reduced in the NAc of chronically stressed mice, and this expression is restored and further increased with antidepressant treatment. Consistent with this, mice lacking *Hdac5* exhibit enhanced depressive-like behaviors in response to chronic stress (Renthal et al., 2007). In the hippocampus, however, chronically stressed mice have increased *Hdac5*, and this can be reversed by antidepressant administration (Tsankova et al., 2006). It is no surprise then that HDAC inhibitors, which have been commonly used as anti-cancer agents, are now also being studied for their antidepressant actions (Eckschlager et al., 2017). For example, MS-275 delivery to the hippocampus reverses anhedonia and reduces social avoidance in mice that experienced continuous social defeat stress (Covington et al., 2011). While HDAC inhibitors remain strong candidates for potential therapeutics in humans, translatability from mouse studies is currently lacking. A gap in this research includes determining whether HDAC expression in one particular brain region may drive MDD; and if so, whether there are therapeutics that may regulate this.

### MicroRNAs in MDD

Several studies have begun to look at miRNAs as a putative peripheral biomarker for MDD. Remarkably, evidence supports that under certain conditions, miRNAs expressed in the brain can cross through the blood–brain barrier and circulate in the plasma (Sheinerman and Umansky, 2013). In patients with MDD, BDNF levels were found to be decreased in plasma (Molendijk et al., 2014). More importantly, two miRNAs known to interact with BDNF have also been found in plasma of MDD individuals (Fang et al., 2018). This study compared the levels of BDNF, miR-132 and miR-124 in MDD patients that were either treated or not treated with citalopram to healthy control patients. It was found that miR-132 was highest in non-treated MDD patients relative to treated patients and controls, suggesting that miR-132 could be used as a potential biomarker for MDD individuals. Notably, miR-132 is the only miRNA that has been consistently identified in several MDD studies (Yuan et al., 2018). Additionally, MDD patients had higher levels of miR-124, with citalopram treated patients having the largest increase (Fang et al., 2018). Conflicting evidence has been reported with regard to how reliable miR-124 plasma expression levels are for being used as an MDD biomarker (Bocchio-Chiavetto et al., 2013; He et al., 2016). Many other prospective miRNA biomarkers have been proposed (Lopez et al., 2018; Yuan et al., 2018), however; much work remains in validating if any of these biomarkers can be used reliably.

Antidepressant drugs are the most common treatment for individuals with MDD, however; many patients do not respond to them. An interesting area of research is focusing on how miRNAs can help predict patient response to antidepressants. Selective serotonin reuptake inhibitors (SSRIs) are a commonly prescribed class of antidepressants that target the serotonin transporter (SERT). Interestingly, it was found that long term treatment of MDD with SSRIs increases the expression of miR-16, which serendipitously also directly targets SERT (Baudry et al., 2010). Subsequently, SSRI promotes the conversion of precursor miR-16 into its mature form to regulate SERT uptake of serotonin. Another study examined the expression of three miRNAs: miR-1202, miR-135a and miR-16, of MDD patients and controls from two independent cohorts and compared miRNA expression between antidepressant responders and non-responders (Fiori et al., 2017). In both cohorts, decreased levels of miR-1202 correlated with patients responding to either an SSRI or a serotonin-norepinephrine reuptake inhibitor (SNRI). After 8 weeks of antidepressant treatment, the responders’ miR-1202 expression levels increased and were indistinguishable from non-responders and the healthy controls. Importantly, *in vitro* studies demonstrated a similar result, where NPCs treated with SSRI drugs had an increase in miR-1202; however, miR-1202 expression did not increase when NPCs were treated with non-serotonergic drugs (Lopez et al., 2014). This suggests that MDD patients with low miR-1202 may be more likely to respond to serotonin-based antidepressants. With continued research, miRNAs may become valuable tools for developing a personalized treatment plan, increasing the chances of patients receiving the most appropriate antidepressant the first time.

In summary, epigenetic studies will be highly beneficial in the development of individualized MDD therapeutics, categorization of MDD subtypes and for enhancing efficacy of currently existing treatments.

## Autism Spectrum Disorders

Autism spectrum disorders are characterized as heritable neurodevelopmental disorders in which affected individuals have deficits in social interactions, communication and behaviors (American Psychiatric Association, 2013). Over the years, genomic studies have identified genes that seem to contribute to the condition (Abrahams and Geschwind, 2008); however, none significantly stand out as a major contributor to ASD. Rather, it appears that much of the heritability is polygenic with each gene only contributing a very small portion. Recent studies are beginning to suggest that in addition to genetics, ASD may also have an epigenetic component.

### Putative Role for Polycomb Repressive Complex 1 in ASD

Several putative genes have been proposed for contributing to ASD, one of which is autism susceptibility candidate 2 (*AUTS2*) (Sultana et al., 2002; Oksenberg and Ahituv, 2013). Surprisingly, recent studies have demonstrated that AUTS2 can be in complex with PRC1 and function in gene promotion contrary to PRC1’s traditional repressive role (Gao et al., 2012; Gao et al., 2014). It is proposed that the PRC1-AUTS2 complex can promote gene expression through the recruitment of CK2 and the co-activator P300 protein. CK2 inhibits monoubiquitination of lysine 119 on histone H2A by phosphorylating RING1B. Further supporting the role of AUTS2 in gene activation, ChIP-seq analysis has localized AUTS2 predominantly near TSSs in the mouse brain. These binding sites also possess active histone marks such as histone 3 lysine 27 acetylation (H3K27ac) and H3K4me3 and were reduced for repressive histone mark H3K27me3. Furthermore, gene ontology analysis of PRC1-AUTS2 targets identified functional terms that were associated with CNS transcriptional programming. All of this evidence supports the PRC1-AUTS2 complex as being involved in promoting gene expression. Behavioral and developmental analysis of *AUTS2* knockout mice also showed similar impaired developmental phenotypes as observed in humans with a disruption in *AUTS2* (Gao et al., 2014). The interaction between AUTS2 and epigenetic machinery could be a rich area to investigate to uncover potential therapeutic targets for individuals with *AUTS2* polymorphisms.

### Differential DNA Methylation

The *SHANK3* gene has been identified as a strong contributing factor to ASDs (Durand et al., 2007; Moessner et al., 2007; Gauthier et al., 2009). In neuronal synapses, SHANK3 acts as a scaffolding protein with critical roles in the formation, maturation and maintenance of synapses (Du et al., 1998; Boeckers et al., 1999). The *SHANK3* gene contains 5 CpG islands at putative intragenic promoters whose methylation status has been associated with alternative splice variants (Zhu et al., 2014). In postmortem ASD brains, there was a significant increase in DNA methylation at the CpG islands 2, 3, and 4 of *SHANK3*. In addition, the methylation at these islands was associated with decreased expression and decreased alternative splicing of *SHANK3*, suggesting DNA methylation regulates the expression of the splice variants. This evidence introduces the possibility that the methylation status of *SHANK3* could serve as a putative predictor for ASD.

### Dysregulation of Non-coding RNAs

Although very little is known about the contributions miRNA and lncRNA make in ASD, several studies have investigated non-coding RNAs in this disorder. One study identified 28 differentially expressed miRNAs in ASD cerebellar cortex tissue using qPCR (Abu-Elneel et al., 2008). Interestingly, 7 of the identified miRNAs were predicted to target autism-associated genes *NEUREXIN* and *SHANK3*. Another study that looked at lncRNA detected over 200 differentially expressed lncRNAs in ASD (Ziats and Rennert, 2013). Of those identified, more than 90% mapped within 500 Kb of a known gene, many of which were genes with functional roles in neurodevelopment and psychiatric diseases. These findings imply that lncRNAs could be part of the mechanism that regulates genes contributing to ASD. This study was also able to compare the expression of lncRNAs in in the cerebellum and cortex from the same patient of healthy and ASD diseased brains. Between brain regions, the ASD brains had significantly less differentially expressed genes and lncRNAs compared to the control brains. This finding is consistent with imaging studies that show autistic brains have less specialized, less distinct regions as compared to healthy brains (Minshew and Keller, 2010). In summary, because ASD lacks a strong heritability factor, epigenetic studies will likely fill in many gaps of mechanisms and risk factors contributing to ASD.

Hundreds of genes have been found to be associated with ASD including *Pcdh* genes. A GWAS study identified 5 SNPs in the *PCDHA* gene that were significantly associated with ASD (Anitha et al., 2013). Interestingly, deletions near *PCDH10* have consistently been found in families with autism (Morrow et al., 2008; Bucan et al., 2009). Further supporting roles for protocadherins in ASD was the finding that ASD brains have increased dendritic spine densities compared to controls (Hutsler and Zhang, 2010). *Pcdh* genes are renowned for their roles in dendritogenesis, dendrite arborization and dendritic spine regulation (Keeler et al., 2015) making them perfect candidate genes for autism studies. Studying *Pcdh* genes in neuropsychiatric diseases has become a hot topic for the field, and it would be interesting to see how epigenetic regulation of them also contributes to disease pathology.

## Fragile X Syndrome

Fragile X syndrome is the most commonly inherited form of mental retardation, and is caused by a trinucleotide repeat in the 5′UTR of the *FMR1* gene, which encodes the RNA binding protein FMRP (Webb et al., 1986; Verkerk et al., 1991; Ashley et al., 1993). FMRP is widely expressed in fetal and adult tissues with the highest enrichment in the brain and testes (Devys et al., 1993). It predominantly localizes in the cytoplasm; however, it can be transported to the nucleus via its nuclear localization signal (Devys et al., 1993; Eberhart et al., 1996). As an RNA binding protein, FMRP appears to have several functions ranging from translation regulation, miRNA-mediated translation suppression and neuronal synaptic plasticity (Jin et al., 2004a). Currently, the precise mechanisms by which FMRP regulates transcription/translation as well as its target RNAs are still under rigorous investigation. Recent work has started to unveil putative functional roles of FMRP as well as potential regulatory targets of FMRP in FXS and other intellectual disabilities (Nelson et al., 2013).

### Hypermethylation of FMR1 Putatively Mediated by RNAi

Fragile X has been shown to be caused by the loss of *FMR1* gene expression in conjunction with the hypermethylation of the cytosines in the CGG trinucleotide repeat (Bell et al., 1991; Pieretti et al., 1991; Sutcliffe et al., 1992; Orsini and Maren, 2012). Methylation of the CGG repeats was identified in human fetal tissue, suggesting that the methylation is acquired after fertilization, or is already present in the carrier female’s oocytes (Sutcliffe et al., 1992). Remarkably, *FMR1* gene expression could be rescued *in vitro* by utilizing DNMT inhibitors and CRISPR/Cas9 to remove the DNA methylation (Bar-Nur et al., 2012; Park et al., 2015; Liu et al., 2018). The question that remains at large is what initiates or causes the hypermethylation of the expanded repeats seen at the CpG island? One model proposes that the RNA interference (RNAi) pathway may be involved (Jin et al., 2004a). This model suggests that the mRNA produced from the expanded *FMR1* gene can fold back on itself, generating a hairpin-like structure and be processed by the RNAi machinery. Ultimately, targeting of the RNAi complex is thought to recruit *de novo* DNMTs and histone methyltransferases (HMTs) to the expanded *FMR1* sequence. This model is supported by the initial finding that the mutant *FMR1* RNA sequence forms different hairpin structures with the prominent structure forming in the 3′UTR of the transcript (Handa et al., 2003).

### MicroRNA Pathway in Fragile X

FMRP has been shown to function as a translational repressor through its RNA binding properties (Laggerbauer et al., 2001; Li et al., 2001). In the brain, FMRP bound to mRNA has been found at dendritic spines associated with polyribosomes, suggesting some involvement in protein synthesis at synapses (Feng et al., 1997). Also, in human brains of fragile X patients there is abnormal dendritic spine growth (Hinton et al., 1991). Recent work has prompted a model where FMRP regulates its mRNA expression through the miRNA pathway. Immunoprecipitation studies demonstrated that mammalian wildtype FMRP, but not mutant FMRP, could associate with miRNA and miRNA pathway proteins Dicer, eIF2C2 and the mammalian Argonaute (AGO) protein (Jin et al., 2004b). This study also determined that in fly, AGO1 is required for dFmr1, the fly ortholog of FMR1, regulation of synaptic plasticity. These observations are supported from previous studies in *Drosophila* that showed *dFmr1* associated with *AGO2* and the RNA inducing silencing complex (RISC) (Caudy et al., 2002; Ishizuka et al., 2002). *In vitro* rescue studies of *FMR1* have demonstrated that current technologies, such as DNMT inhibitors and CRISPR/Cas9, can be applied as putative therapeutics. The next step is to conduct translational studies to test whether *FMR1* expression can be rescued in mammals. A possible place to begin would be *in vitro* fertilization experiments. Hypermethylation of *FMR1* is observed either after fertilization or is already in the oocyte. It would be interesting to explore the effects of CRISPR technology on *FMR1* expression at these early stages in development.

## Rett Syndrome

### MeCP2 Dysregulation

Rett syndrome (RTT) is a rare disease that was first described in 1966 although the criteria for diagnosing patients did not become available until the 1980s (Rett, 1966; Hagberg et al., 1985). Described as a progressive neurodevelopment disorder, Rett syndrome is most common in females and symptoms, such as autistic behavior, stereotypic hand wringing and loss of facial expression, begin to appear around 18 months of age (Hagberg et al., 1983). Later clinical presentations can include difficulty with motor control, breathing, communication, small head size, muscle wasting and seizures (Gold et al., 2018). The Rett loci was mapped to a region on the X chromosome (Xq28) (Sirianni et al., 1998). Further mapping studies found that the *MeCP2* gene also mapped to this region, and that mutations in the methyl binding domain and transcription of repression domain (TRD) of MeCP2 caused RTT (Amir et al., 1999). The *MeCP2* missense mutation R133C results in the abolishment of any methyl binding ability of the MBD (Mellen et al., 2012). Many other RTT-associated missense mutations in the MBD and TRD also have been shown to prevent MeCP2’s ability to interact with complexes and methylated DNA (Lyst et al., 2013). MeCP2 is essential to normal brain morphology and consequently, individuals with RTT have more densely packed, shorter neurons with dendrites that are less dense and less complex (Armstrong et al., 1995). Conditional deletion of *Mecp2* in postnatal mice produced similar phenotypes as those observed in RTT patients (Gemelli et al., 2006). These mice had impaired motor coordination, increased anxiety and abnormal social behavior.

The mechanism by which *MeCP2* mutation (or loss of function) causes Rett is not known; although several studies have tried to identify dysregulated genes in RTT that are direct targets of MeCP2 (Colantuoni et al., 2001; Peddada et al., 2006; Jordan et al., 2007). One study determined that *MeCP2* deficient mice and RTT human brains showed significant upregulation of inhibitors of differentiation genes (ID1-4), which are targets of MeCP2 (Peddada et al., 2006). *In vitro* studies demonstrated that MeCP2 normally downregulates the protocadherin genes *PCDHB1* and *PCDH7* (Miyake et al., 2011). Because protocadherins are critical for proper brain development, aberrant expression of these genes could contribute to the pathogenesis of RTT. Furthermore, a study involving four independent cDNA microarrays demonstrated that the majority of differentially expressed genes were downregulated in human RTT postmortem brains, but they failed to investigate whether any of these genes were associated with MeCP2 (Colantuoni et al., 2001). Another study that used microarrays to identify differentially expressed genes in a *MeCP2-null* mouse model looked at several brain regions (cortex, midbrain and cerebellum) to determine if certain regions were more sensitive to loss of *MeCP2* (Urdinguio et al., 2008). Although no significant differences were found between brain regions, the study did identify several genes that are direct binding targets of MeCP2. Importantly, these genes (*Fkbp5, Mobp, Plagl1, Ddc, Mllt2h, Eya2*, and *S100a9*) were found to be upregulated in the RTT mouse model, and their functions are associated with neural function. Identifying candidate genes in RTT is important for developing a greater understanding of the underlying mechanism.

While Rett syndrome is the result of *MeCP2* loss of function, MeCP2 duplication syndrome is the result of *MeCP2* overexpression, and mimics some of the symptoms of Rett (Van Esch et al., 2005). Rodent models have clearly demonstrated that having a balance of *MeCP2* expression and function is absolutely essential to normal brain activity. Mice that have either overexpression or deletion of *MeCP2* show disrupted neuronal activity in the hippocampus (Lu et al., 2016). Both mouse models exhibit neuronal hypersynchrony, which is an aberration from the normal asynchrony typically present at baseline. Importantly, this phenotype could be observed several months before the animals started to have seizures. Deep brain stimulation therapy rescued the abnormal synchrony in both mouse models. Thus, proper *MeCP2* expression levels are required for stable neuronal activity.

### DNA Methylation Affects MeCP2 Binding

Several studies have looked at how dynamic changes in DNA methylation, of both CpG and CpH, could correlate to Rett syndrome pathology, and have even speculated as to how they could contribute to the delayed onset of RTT symptoms. In addition to mCG, MeCP2 also binds to mCH, preferentially to mCA (Guo et al., 2014; Gabel et al., 2015). Accumulation of MeCP2 in mammalian neurons occurs early after birth when mCH starts to accumulate (Shahbazian et al., 2002; Ballas et al., 2009; Lister et al., 2013). Interestingly, in maturing neurons, those genes that acquired mCH marks were more likely to be dysregulated in the RTT mouse model (Chen et al., 2015). This evidence advocates that early in brain development, MeCP2 initially binds to mCG and then to mCH as it accumulates around a subgroup of neuronal genes (such as *Bdnf*) to influence gene transcription. This epigenetic mechanism could contribute to why a genetic disease like Rett syndrome could have a delayed onset.

Important for brain development is the proper regulation of LINE 1 retrotransposon. MeCP2 directly targets the 5′UTR of LINE 1 in the brain to regulate LINE 1 mobility (Muotri et al., 2010). Mutations in *MeCP2*, as seen in RTT, prevent its binding to LINE 1 resulting in increased expression of LINE 1 in both *in vitro* and *in vivo* models of RTT. Whether or not the increase in neuronal retrotransposition contributes to the cause of RTT, or is simply an effect is not clear. These findings warrant further investigation of LINE 1’s contribution to RTT. It could also be worth investigating global methylation profiles of developing mouse embryos to determine if DNA methylation patterns are also disrupted, contributing to aberrant LINE 1 expression.

### MeCP2 Interacts With HDACs

A possible epigenetic mechanism to investigate for RTT is the interaction of MeCP2 with HDAC3. MeCP2 associates with HDAC3 as part of the NCoR/SMRT co-suppressor complex (Lyst et al., 2013), and *MeCP2* missense mutations that occur in Rett Syndrome prevent this interaction (Nan et al., 1998; Ebert et al., 2013; Lyst et al., 2013). Furthermore, in mice, HDAC3 binds near transcriptional start sites of active gene promoters, including the *Bdnf* gene promoter in the brain (Nott et al., 2016). In Rett syndromic mice, *MeCP2* mutations prevent the recruitment of HDAC3 and FOXO to gene promoters. FOXO is a transcription factor that when acetylated has reduced binding affinity to DNA (Daitoku et al., 2004; Matsuzaki et al., 2005; Hatta et al., 2009). Recruitment of HDAC3 to active gene promoters through MeCP2 regulates the deacetylation of FOXO, and promotes gene expression of neuronal genes (Nott et al., 2016). The gene targets of this complex might yield insightful avenues for developing site-directed therapeutics for Rett patients.

### Dysregulation of miRNAs

Recently, miRNAs have been suggested to interact with MeCP2 and potentially contribute to RTT. Using a mouse RTT model, one study found that just over one-fourth of the miRNAs analyzed showed different expression patterns in *Mecp2*-null brains compared to wildtype, most of which were downregulated (Urdinguio et al., 2010). Additionally, they found that MeCP2 associated with the miRNAs that had 5′UTR hypermethylation. Interestingly, two of the downregulated miRNA, miR-146a and miR-146b, base pair to the 3′UTR of IL-1 receptor-associated kinase 1 (Irak1), which is upregulated in RTT mouse brains (Taganov et al., 2006; Urdinguio et al., 2008). It was then shown that both miR-146a and miR-146b could downregulate *IRAK1* expression *in vitro* (Nahid et al., 2009) and it was proposed that in Rett syndrome the downregulation of miR-146a/b contributes to the overexpression of *IRAK1* (Urdinguio et al., 2010). Another study identified altered expression of miRNA in the cerebellum of *Mecp2*-null mice (Wu et al., 2010b). They showed that the promoters of the dysregulated miRNAs were methylated and bound by MeCP2, downregulating their expression. Furthermore, the 3′UTR of the *Bdnf* transcript contained multiple miRNA binding sites for miRNA that were upregulated, providing mechanistic evidence to explain reduced *Bdnf* expression in RTT. MeCP2 and its interactions with epigenetic factors play major roles in Rett syndrome, yet why there is a delay in disease onset is not fully elucidated. It may be worthwhile to investigate a spectrum of early developmental stages to determine what epigenetic changes are occurring before the onset of disease, and how these changes could contribute to the delayed onset of RTT.

## Schizophrenia

Schizophrenia (SZ) is a mental illness with clinical phenotypes such as dissociation of thought, ideas, identity and emotion (Moskowitz and Heim, 2011). SZ has a wide range of first episodic onset, with early onset occurring in adolescence and late onset being in the mid-50s (Jablensky et al., 1992). Interestingly, males appear to experience their first episodic event 4–5 years earlier than females, on average (Hafner et al., 1998). Like ASD, SZ lacks a single causal gene (International Schizophrenia et al., 2009); however, epigenetic factors are a promising area of research.

### Aberrant DNA Methylation

In all postmortem brains of schizophrenic individuals, studies have found ∼50% increase in DNA methylation at the *Reelin* gene (*RELN)* promoter (Impagnatiello et al., 1998). Reelin is an extracellular matrix protein highly expressed in GABAergic neurons (Pesold et al., 1999). Functionally, RELN has been shown to be essential in brain development contributing to neuronal migration, axonal branching and synaptogenesis. Upstream of the promoter region is a CpG island, suggesting that inappropriate methylation could regulate *RELN* (Royaux et al., 1997). One study demonstrated that hypermethylation of the *RELN* promoter was associated with a decrease of *RELN* expression found in the brains of schizophrenic patients (Abdolmaleky et al., 2005). It was also established that the transcription factors Sp1 and Tbr1 have binding sites upstream of the *RELN* promoter and induce gene expression (Chen et al., 2002). Interestingly, Sp1 regulation of the adenine ribosyltransferase gene triggers demethylation and prevents *de novo* methylation, and it is proposed that Sp1 could similarly regulate *RELN* (Han et al., 2001; Chen et al., 2002). Additionally, prevention of DNA methylation with 5-aza-2′-deoxycytidine (5-azadC) at the CpG island increased gene expression of *RELN* more than 50-fold (Chen et al., 2002). This evidence indicates that methylation at the *RELN* gene likely plays a major role in SZ.

Very little research has been done to link how aberrant epigenetic modifications can affect the expression of protocadherins in SZ. Interestingly, olanzapine, a common antipsychotic drug often prescribed to SZ patients, is proposed to induce its effect by causing DNA methylation changes throughout the brain (Melka et al., 2014). Importantly, several protocadherin genes (*Pcdha11*, *Pcdha9*, and *Pcdhga5*) had altered promoter methylation in the cerebellum, whereas hypomethylation of the *Pcdhga8* promoter was observed in the hippocampus. Regions of the genome that are thought to contribute to SZ susceptibility appear to overlap with cadherin superfamily genes (Pedrosa et al., 2008). Polymorphisms in *PCDH12* and *PCDH15* were found to be in association (Gregorio et al., 2009; Narayanan et al., 2015), and linkage studies found the *CTNNA2* gene in sibling pairs with SZ (DeLisi et al., 2002; Chu and Liu, 2010). Protocadherin gene expression clearly has important roles in SZ, but to what extent they are affected by epigenetic changes is unclear. It would be interesting to test whether manipulation of methylation at various protocadherin genes could significantly impact brain development and function in neuropsychiatric diseases.

### Non-coding RNAs

The contribution of miRNAs to cognitive disorders has been best characterized in SZ. In postmortem SZ brains, miR-132 was found to be dysregulated, and has been associated with cognitive and behavioral impairments (Moreau et al., 2011; Miller et al., 2012). In the prefrontal cortex of SZ brains, miR-132 was significantly downregulated while its target mRNAs were all upregulated (Miller et al., 2012). Some of the identified targets were associated with synaptic long-term potentiation and depression, neuronal CREB signaling and DNA methylation. Interestingly, *Dnmt3a* was found to be a putative target of miR-132; however, the expression patterns of *Dnmt3a* and miR-132 at early developmental stages are opposite. It is not until later in development when miR-132 expression drastically increases that it would have the potential to target *Dnmt3a*. One could speculate that this temporal expression of miR-132 and Dnmt3a prevents their dysregulation during early development, consistent with SZ requiring an ongoing and prolonged accumulation of dysregulated events that must reach a threshold for symptoms to develop (Cannon, 1996). Another miRNA found in SZ brains was miR-195, which targets several genes (*BDNF*, *RELN*, *DRD1*) implicated in SZ (Beveridge et al., 2010). GWAS for SZ have identified a locus on chromosome 1p21.3 that is highly associated with miR-137 (Schizophrenia Working Group of the Psychiatric Genomics Consortium, 2014; Gianfrancesco et al., 2017). Several independent GWAS studies have identified a single nucleotide polymorphism (SNP) within the miR-137 gene that is common amongst schizophrenic patients (Hamshere et al., 2013; Guan et al., 2014). Patients with this high-risk SNP had earlier age of onset (Lett et al., 2013), abnormal development of brain structure and lower prefrontal cortex activity during working memory (van Erp et al., 2014). *In vitro* analysis identified a novel lncRNA whose expression pattern is very comparable to miR-137 (Gianfrancesco et al., 2017). The lncRNA was found to be highly expressed, specifically in the prefrontal cortex, and transcriptionally induced by psychoactive drugs, suggesting that there might be a potential connection with the hallucinations that many SZ patients experience. Although further work still needs to be done to better understand the functional role of this lncRNA, it could be postulated that miR-137 and the lncRNA could regulate each other.

Over 200 lncRNAs have been found in the brains of individuals with psychiatric disorders such as SZ (Ziats and Rennert, 2013). The lncRNA *GOMAFU* in humans is involved in brain development (Mercer et al., 2010) and post-mitotic neuronal function (Sone et al., 2007). In addition, it has been found that SZ patients have reduced *GOMAFU* expression, which was found to be important for cognitive function (Barry et al., 2014). Interestingly, *GOMAFU* can directly interact with the splicing factors quaking (QKI) and SRSF1 (serine/arginine-rich splicing factor 1), and when *GOMAFU* is dysregulated, the alternative splicing resembles that seen in schizophrenia-associated genes *DISC1* and *ERBB4* (Figure 1D). QKI was identified as a potential SZ gene because it is the only gene located in the chromosome susceptibility locus, 6q25-6q27, in a schizophrenia pedigree (Aberg et al., 2006b). mRNA expression analysis revealed that two QKI splice variants were significantly down regulated in SZ patient brains, suggesting that the splice variants could increase the susceptibility of SZ. Moreover, disrupted QKI splicing could account for the decreased expression of myelin-related genes associated with SZ (Aberg et al., 2006a). Interestingly, most of the myelin-related gene repression was explained by the splice variant QKI-7kb, and putative QKI-binding sites were identified in five myelin gene’s mRNA.

In summary, non-coding RNAs as well as methylation of the *RELN* gene have been implicated as epigenetic research areas that may hold potential therapeutic targets for SZ. Future studies should aim to further elucidate the role of miRNAs in the SZ brain, in order to pinpoint certain miRNAs that may be pivotal in SZ symptoms. Because SZ has so many genetic variants that only contribute a small portion to the overall increased risk, identifying global epigenetic dysregulation patterns may be more promising. Additionally, there is a lack of studies looking at how epigenetic patterning in the brain changes due to environmental risk factors such as drug use, birth complications and childhood adversity (Neilson et al., 2017). All of these environmental risk factors have been highly correlated with SZ and shown to impact brain development.

## Conclusion and Outlook

Genetic and epigenetic regulations are critical for brain development, function and prevention of neurological diseases. Currently, the field lacks clear molecular mechanisms underlying neuropsychiatric diseases and effective treatment options. Epigenetics provides a whole new dimension for therapeutic treatments because so many of these diseases are not monogenic and likely have a significant environmental contribution. The epigenome is greatly influenced by environmental factors such as nutrition, chemical pollutants, traumatic early life experiences, temperature changes and exercise (Roth and Sweatt, 2011; Feil and Fraga, 2012), but how they affect brain development is poorly understood. Importantly, the effect of the environment on epigenetics is not limited to development after birth, but can also affect development *in utero*. Recent work hypothesized that early life stressors that cause long-lasting epigenetic changes may be due to cellular epigenetic “priming.” Similar to the immune system, once a particular environmental exposure is experienced and alters the epigenetic state of a gene, that gene now remains in a state of “primed responsiveness,” and will have a quicker response if that same environmental exposure is experienced again (Vineis et al., 2017). This concept of epigenetic memory in response to environmental stimuli could serve as a way to identify individuals predisposed to developing neuropsychiatric diseases.

For several of the monogenic neuropsychiatric diseases, such as Rett syndrome and Fragile X, exploring epigenetic mechanisms may lead to understanding whether or not there could be early intervention treatment that could attenuate the disease prior to its onset. Prenatal genome sequencing could be implemented to look for mutations in specific genes as the cost of sequencing continuously decreases. If it is known ahead of time that a child is predisposed, early intervention treatments could be started to slow or prevent disease progression. Possible directions for treatment development could include the use of CRISPR editing to fix missenses mutations in MeCP2 of Rett patients, or developing DNMT based drugs to remove the methylation on the CGG expanded repeat in Fragile X. Additionally, it could be useful to look at developmental time points to identify what epigenetic changes are occurring just before the onset of disease. This could shed light on when key epigenetic remodeling events take place and when potential interventions could be tested.

Treatments for polygenic neuropsychiatric diseases, such as MDD, could benefit the most from epigenetic treatments because there is no clear-cut mechanism to explain disease development. The field is currently focusing on exploring two approaches for developing HDAC inhibitor treatments. The first approach combines HDAC inhibitors with antidepressant drugs (Fuchikami et al., 2016). In this method, HDACs are thought to promote the condensation of the chromatin and prevent transcription factors from binding, regardless of whether the antidepressant is able to increase the levels of the transcription factor. Administration of both HDAC inhibitors and antidepressant could make both drugs work better. The second approach addresses the problem of low specificity of current HDAC inhibitors. The goal of this approach is to synthesize new, highly selective compounds/analogs that can cross the blood brain barrier and be administered acutely instead of chronically (Misztak et al., 2018). Additionally, identifying more reliable biomarkers, such as miR-1202, that can help predict a patient’s likelihood to respond to antidepressants could eliminate much of the guess work in finding a drug that will best treat a patient.

In summary, this review has discussed several epigenetic processes and how dysregulation of any of them can affect brain development, function and disease. An important topic not covered in this review is that dysregulation of DNA methylation, histone modifications, chromatin remodeling, and regulatory RNA also contribute to neurodegenerative diseases such as Huntington’s, Parkinson’s and Alzheimer’s. Several model systems, such as mice and postmortem human brains, have been used to generate the current knowledge bank available. A promising new model system, the organoid, can help evolve our understanding of genetics and epigenetics in neuropsychiatric disorders.

Currently, a major challenge in studying neuropsychiatric diseases is the limitations of the model systems available. Mouse models and human postmortem brains have been heavily relied upon to provide insight into neuropsychiatric disease pathology and etiology. However, both options have their limitations. Although mouse and human brains are highly similar at genetic, structural and general circuitry levels, key differences limit them as models of human diseases that are characterized by complex dysfunction of behavior and thought. For example, human brains have evolved to contain the granular prefrontal cortex, which is absent in mouse brains (Passingham and Wise, 2012). This portion of the cortex is thought to have emerged in relation to increasing brain size, and have roles in comprehension, planning and perception (Goldman-Rakic, 1996; Barbas, 2000; Rolls, 2000; Miller and Cohen, 2001). Human brain samples are obtained postmortem, and thus can never fully recapitulate the epigenetic landscape of a living brain. Postmortem brains only provide a snap shot in the timeline of the disease, and this snapshot is usually biased toward the state of death. Thus, postmortem human brains fail to provide data regarding disease initiation and progression over time.

A new and promising model system that can compensate for animal models and postmortem brains are organoids. Organoids are 3-dimensional cultures that model whole developing organs (Itskovitz-Eldor et al., 2000). This system evolved from embryoid cultures, which are 3D aggregates of stem cells that are grown in a suspension that will induce their differentiation. When organoids are used to generate neuronal lineages, they can recapitulate human brain development *in vitro*. Morphological studies have further confirmed that forebrain organoids have similar developmental patterns as the developing human cortex (Qian et al., 2016; Zhang et al., 2016). For example, developing organoids can undergo neural differentiation, form multi-layer progenitor zones, form discrete brain regions and portray typical neuron morphologies such as spine-like structures (Lancaster et al., 2013; Qian et al., 2016). Epigenomic studies have also confirmed that brain organoids recapitulate the fetal brain epigenome (Luo et al., 2016). Whole-genome methylome profiling revealed that mCH accumulation in both fetal brain and cerebral organoid occurred at super-enhancers that are specifically active during fetal development, and later became repressed. Additionally, organoid mCG signatures at DNA methylated valleys, large domains depleted of mCG, were comparable to fetal cortex and localized to genes with roles in brain development. Organoids are cultured from mature epithelial cells that are reverted back to induced pluripotent stem cells. The mature epithelial cells can be obtained non-invasively from an individual affected by a neurological disease, allowing researchers to use a model that is genetically identical to the patient. This provides the field with the ability to develop unique therapeutic options specific to each patient.

In conclusion, the study of epigenetics, along with the exploitation of organoid models, can accelerate our understanding of neuropsychiatric diseases to better develop enhanced treatments.

## Author Contributions

JK and BY wrote the review together. EB and ZW helped with revisions. EB contributed to the MDD section.

## Conflict of Interest Statement

The authors declare that the research was conducted in the absence of any commercial or financial relationships that could be construed as a potential conflict of interest.
